# Neonative Diploid-Polyploid Hotspots of *Paspalum notatum*: Identifying Novel Genetic Diversity for Conservation in South America

**DOI:** 10.3390/genes16091098

**Published:** 2025-09-16

**Authors:** Lucas M. Escobar, Anna Verena Reutemann, María C. Perichon, Juan S. Schneider, Carolina A. Sartor, Clarisse Chaparro, Julio R. Daviña, José F. M. Valls, Eric J. Martínez, Ana I. Honfi

**Affiliations:** 1 Programa de Estudios Florísticos y Genética Vegetal, Instituto de Biología Subtropical (CONICET-UNaM), Facultad de Ciencias Exactas Químicas y Naturales, Universidad Nacional de Misiones (FCEQyN-UNaM), Posadas 3300, Argentina; lucasmescobar17@gmail.com (L.M.E.); constanzaperichon@gmail.com (M.C.P.); schneider.s.juan@gmail.com (J.S.S.); caro.sartor@hotmail.com (C.A.S.); juliordavina@gmail.com (J.R.D.); 2 Laboratorio de Genética y Mejoramiento de Especies Forrajeras, Instituto de Botánica del Nordeste CONICET-UNNE, Facultad de Ciencias Agrarias, Universidad Nacional del Nordeste (FCA-UNNE), Corrientes 3400, Argentina; vreutemann@gmail.com (A.V.R.); eric@agr.unne.edu.ar (E.J.M.); 3 Facultad de Ciencias Exactas y Naturales, Universidad Nacional de Asunción, San Lorenzo 170901, Paraguay; cchaparro@cepbuna.edu.py; 4 EMBRAPA Recursos Genéticos e Biotecnología, Brasilia 70770-917, DF, Brazil; jose.valls@embrapa.br

**Keywords:** chromosome number, cytotype diversity, neopolyploidization, wild-cultivated plant hybrids, cytotype foci conservation

## Abstract

**Background**: Bahiagrass (*Paspalum notatum*), a key cultivated grass worldwide, includes both sexual diploid and apomictic tetraploid cytotypes. Finding new diploid populations is crucial for the species’ genetic improvement and conservation. **Objectives**: We aimed to determine the ploidy levels of 168 *P. notatum* accessions from subtropical South America, analyze the geographic distribution of cytotype diversity, and identify new diploid zones. **Methods**: Using chromosome counts and flow cytometry, we georeferenced our data with existing literature to map cytotype distribution. **Results**: We discovered five previously unknown diploid centers in Argentina, Brazil, and Paraguay, two of which resulted from the naturalization of diploid cultivars. One location hosted a mixed-ploidy population (diploid, tetraploid, and pentaploid), confirming ongoing hybridization. Our results show that human activity actively creates new centers of genetic diversity, serving as a dynamic source of raw material for crop resilience. These neonative diversification zones are not only of scientific interest but also vital, evolving hotspots for germplasm conservation. **Conclusions**: This study presents a new framework for understanding the interactions between crop and wild relatives and highlights the urgent need for conservation in the rapidly changing South American grasslands.

## 1. Introduction

Natural grasslands of South America are a reservoir of native genetic resources. Livestock farming distributed throughout these grasslands depends on native and cultivated forages, combining productive landscapes with natural biodiversity. Habitat disturbance by anthropogenic activities and biological invasions creates new contact zones between conspecific and heterospecific populations that were previously isolated by distance or topography [1]. For many species, human-induced environmental changes are indirect drivers of species’ latitudinal and altitudinal range contraction or expansion [2]. Range dynamics can help to identify the factors that enable a species to disperse and establish in new areas not previously colonized naturally. While these interactions can threaten local ecotypes, they can also create novel genetic combinations, representing both a conservation challenge and a unique opportunity. Failure to identify and characterize these emerging ‘neonative’ populations may lead to the irreversible loss of valuable genetic resources before they are even recognized.

Contrary to native species, alien species originate from direct anthropogenic action, such as the introduction of cultivated plants [3]. When alien species hybridize with native species or with other alien taxa, they may give rise to a neonative species [2,3]. Thus, alien species could affect local populations by environmental and/or genetic introgression, hybridization, and admixture [4]. The environmental and genetic consequences of these disturbances are usually observed in contact zones of wild relatives and crops [5]. Neonative is a modern term [2] used for range-expanding species that originate from anthropogenic environmental changes. One or several repeated introductions, including escaped cultivated plants, interact with native local surrounding populations of the same species, shaping a new genetic and cytotypic landscape.

The gene pool of a biological species is the sum of all the genes, alleles, and karyotypes shared by individuals of a Mendelian population. Considering cultivated and wild related species, Harlan & De Wet [6] developed a classification system based on relatedness, where the gene pool is characterized by basic chromosome numbers (*x*), ploidies (2n & n), and genome constitution, reflecting the evolutionary trajectories of the involved species. The classification of primary, secondary and tertiary gene pools was applied to several crops and wild relatives [6]. For example, recent reports for the gene pool status of *Arachis* L., *Eleusine* Gaertn., *Oryza* L., *Ipomoea* L., *Solanum* L. were used to design breeding crosses and conservation strategies [5,7].

Due to its forage value and rapid establishment of a dense ground cover, numerous accessions of *Paspalum notatum* Flüggé (Bahiagrass) have been incorporated into agronomic experiments, and abundant commercial cultivars have been released [8,9]. *Paspalum notatum* is a multiploid species with an intraspecific ploidy series comprising 2*x*, 3*x*, 4*x*, 5*x*, and 6*x* cytotypes (*x* = 10) [9,10,11,12]. This species is polymorphic, presenting two botanical varieties differentiated by their ploidy level and morphological characteristics. The most common cytotype of the species, *P. notatum* var. *notatum*, is autotetraploid (2*n* = 4*x* = 40), apomictic, pseudogamous and self-compatible [11,13,14,15,16]. This cytotype is widely distributed from Mesoamerica and the Caribbean Islands to Uruguay, Argentina, Brazil, and Paraguay [17,18]. On the other hand, *P. notatum* var. *saurae* Parodi has a diploid cytotype (2*n* = 2*x* = 20), and is sexually self-sterile [10,19,20]. Diploid Bahiagrass occurs naturally in a restricted area of Argentina, between the Uruguay and Paraná rivers [21,22,23]. This restricted area was proposed as its center of origin [18,21,24]. Bahiagrass is a natural agamic complex, comprising 2*x* + 4*x* cytotypes whose karyotype and genome size indicated autopolyploidy and genome downsizing [12]. Rare triploids [14,25,26,27,28]; and a pentaploid [27] were also observed in this agamic complex.

The diploid form (*P. notatum* var. *saurae*), also known as Pensacola Bahiagrass, occurs naturally in a restricted geographical area of Argentina stretching between the western and eastern banks of the Uruguay and Paraná rivers [21,22,23]. The highly diverse populations in a small area of the province of Santa Fe, on the banks of the Paraná River and the island of Berduc, near the city of Cayastá were key for proposing the center of origin of the species [18,21,24].

Historically, the center of origin of *P. notatum* species was early proposed by Parodi [21,22] as a broad area comprising Paraguay, southern Brazil, northeastern Argentine, and Uruguay, and is considered a meadow grass with morphological polymorphisms among biotypes inhabiting this area [29,30,31]. The most common cultivar of diploid Bahiagrass is cv. Pensacola, which stands out for its easy establishment, trampling persistence, grow habit on sandy soils with sporadic flooding, and seed production [9,12,32,33]. It was accidentally introduced to the United States probably before 1926 [24], from its native geographical distribution—a small area in central-eastern Argentina [18,21,22,24]. Today, several millions hectares of cv. Pensacola are cultivated as forage or turf in many temperate and warm regions around the world [33,34]. Besides cv. Pensacola, other popular diploid bahiagrass cultivars include Tifhi1, Tifton 9, TifQuik, and UF-Riata [34,35,36,37,38,39]. On the other hand, the most popular cultivars of apomictic tetraploid Bahiagrass are Argentine, Paraguay, Paraguay22, Wilmington, “common”, Boyero–UNNE, Tuim, Aruaí, Tiriba and Maritaca [34,35,36,37,38,39].

This species forms the basis of the best natural grasslands in Argentina, Paraguay, Uruguay, and Brazil [21,40]. However, significant gaps persist in our knowledge of *P. notatum*’s cytotype diversity, particularly concerning the interaction between wild populations and escaped cultivars. This lack of information severely hampers our ability to formulate effective in situ conservation strategies and to fully utilize the species’ genetic potential. Research efforts focused on mapping centers of origin and diversity of major domesticated plant species and associated wild relatives [41] have not considered, at microscale, the composition and persistence of anthropogenic neocenters derived of accidental escapes of cultivated plants.

The aims of this work were (i) to determine the chromosome number and ploidy level of new accessions of *P. notatum* from subtropical South America, (ii) to analyze the geographical distribution of cytotype diversity to locate new diploid areas, and (iii) to propose a conceptual framework for the identification and conservation of these neonative cytotypic diversity centers.

## 2. Materials and Methods

### 2.1. Plant Material

One hundred and sixty-eight (N = 168) accessions of *P. notatum* from subtropical South America were studied. Germplasm collections were made, including both botanical varieties, *P. notatum* var. *saurae* and *P. notatum* var *notatum*. The plant material belonged to collections made in different locations in Argentina, Brazil, and Paraguay (Figure S1). The provenances of the studied material are detailed in Table 1. The voucher specimens were deposited at the Herbarium of the National University of Misiones (MNES) in Argentina, Herbarium of the Natural and Exact Sciences Faculty of Asuncion National University (FACEN) in Paraguay, and/or Herbarium of the National Centre for Genetic Resources and Biotechnology (CEN) in Brazil.

Sampling areas were selected based on herbarium specimens of *P. notatum* var. *saurae* collected from the subtropical regions of South America. Special attention was given to specimens clearly situated beyond the typical distribution range of the diploid variety in Argentina (south of Santa Fe). Today, some of these localities have become croplands. All samples were taken from natural populations. Preferably, at least two to several individuals were sampled per population. Individual plants were at least 10 m apart from each other. The sampling sites in Argentina were in subtropical open grasslands. In southern Entre Rios (H1961 accession), the river basin margins of the Uruguay River exhibit considerable development of flood plains and sporadic marshlands and receive substantial allochthonous material from the bordering marginal vegetation and from grassland terrestrial vegetation during flood-pulse periods. The remaining accessions were sampled in open grasslands across Argentina, Paraguay and Brazil, where the species grows extensively as a continuous carpet that completely covers the ground for many kilometers. Some of them occur along roadsides.

Collection sites were categorized into (1) sites for ploidy determination only (up to three individuals were collected) and (2) sites to evaluate cytotype diversity at the population level (at least 7 individuals per population were collected) (Table 1 and Table 2). Overall, samples were collected from 76 localities, of which 3 were selected for population evaluations (H1961, H1740, H2674) (Table 1 and Table 2). A transect spanning the longest length available across the population was followed to obtain information on local dispersal of cytotypes. An even representation of individuals within the population was attained by uniform sampling (i.e., the distance between two consecutive individuals was maintained consistently, and varied between 10 and 12 m depending on the spatial dimensions of each population). To conduct the cytogenetic studies, parts of the rhizomes of the sampled individuals were transplanted into pots and cultivated with GROWMIX-PRO substrate, composed of Sphagnum peat moss of medium and fine fibers, vermiculite, lime calcite, lime dolomite, humidifying agents, sand and soil. All collected plants were grown and maintained under a greenhouse at the Subtropical Biology Institute.

### 2.2. Chromosome Counts

Chromosome counts were performed on young root tips pre-treated with a saturated solution of 1-bromonaphthalene for 3 h and then fixed in ethanol: glacial acetic acid (3:1) for at least 24 h at 4 °C. Fixed root-tips were stored in the fixative at 4 °C until used. Conventional Feulgen staining was performed, for which the rootlets were hydrolysed in 1N HCl for 10 min at 60 °C using a thermostatic bath, followed by staining with basic fuchsine (Schiff’s reagent) [42] in a dark room. Finally, the root tips were macerated using 2% acetic orcein to produce a contrast stain. Semi-permanent slides were prepared by sealing the coverslip with a rubber solution to examine chromosome number and morphology.

### 2.3. Ploidy Analyses by Flow Cytometry

Ploidy levels were also determined by estimating the relative DNA content of leaf tissues by flow cytometry (FC). Ploidy was estimated using the CyStain absolute P kit (Partec) and measured with a CyFlow^®^ Space flow cytometer (Sysmex-Partec, Görlitz, Germany). The protocol of Galdeano et al. [33] and Honfi et al. [12] were used. Briefly, fresh leaves were collected a few hours before use and leaf parts were then selected in the laboratory. In bulk analyses, the leaf samples from up to 5 individuals, including the standard, were placed in a Petri dish, to which 0.5 mL of extraction solution was added. Subsequently, the samples were chopped using a scalpel and left in the extraction buffer for one minute. Following 1 min incubation, samples were filtered through a 50 µm nylon mesh directly into the sample tube, to which 1.5 mL of DAPI (4′,6-diamidino2-phenylindole) stain solution was added. The mixture was incubated for another 2 min at room temperature and then analysed. Relative DNA content was estimated regarding to the standard reference, using individuals of *P. notatum* with known chromosome numbers or the standard for *P. notatum H1961* (2*n* = 2*x* = 20, 1CX = 1.438 pg [12]. Three measures per sample were performed, which were analysed using FloMax Software 2016 v 2.11 (Quantum Analysis Gbmh, 2016). The relative fluorescence of at least 3000 particles (nuclei) was measured for each sample using FloMax (Partec, Münster, Germany) and a maximum coefficient of variation (CV) value of 5% was accepted for each sample peak (G0/G1 peak). Ploidy was estimated by comparing the DNA histogram peaks of the samples with those of their internal standard. The procedures for handling ambiguous results consisted of applying classical chromosome counts for each individual plant. For validation purposes, we also considered three measures per sample, proper cleaning procedures prior to analyzing each sample, and in some cases, measurements were replicated on different dates. In doubtful cases, mitotic chromosome counts were used to verify and validate the results.

### 2.4. Georeferenced Maps

A cytogeographic map was constructed using the chromosome database of the Floristic Studies and Plant Genetics Program (PEFyGV) of the Subtropical Biology Institute (IBS, UNaM-CONICET). A curate list from literature of accessions with chromosome counts was added to the data of the present work (Table S1). Accessions were georeferenced on a South American map. We used R version 4.5.0 (R Core Team, 2024) [43] implementing functions from *sf* [44], *geodata* [45], *ggplot2* [46] and *ggspatial* [47] packages for creating the maps. The projection onto a map is used to illustrate the geographic distribution area occupied by *P. notatum* cytotypes and to identify neodiploid areas.

## 3. Results

### 3.1. Chromosome Number and Ploidy Level

**Figure 1:**
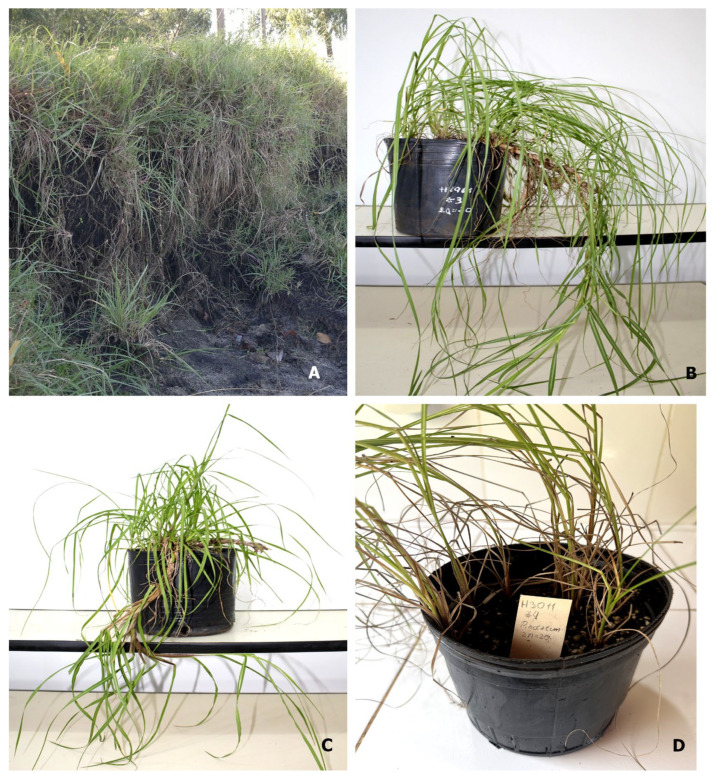
Accession plants of *P. notatum* var*. saurae*. (**A**) H1961 natural population from Entre Ríos, Argentina. Note the riverbank habitat. (**B**) Cultivated individual plant from H1961 accession. Note the vigorous rhizomes. (**C**) Cultivated individual plant from H1740 accession from Itapúa, Paraguay. (**D**) Cultivated individual plant from H3011#4 accession from Santa Catarina, Brazil.

**Figure 2:**
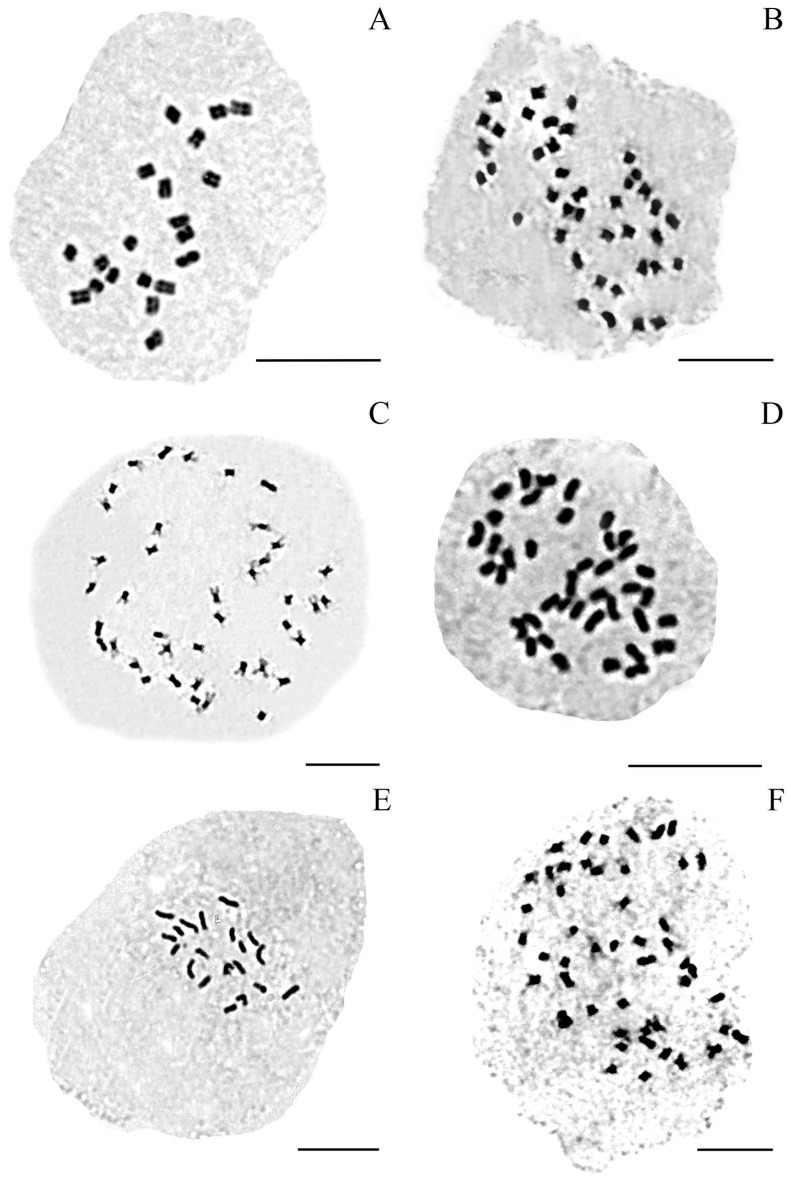
Mitotic metaphases of *P. notatum* cytotypes. (**A**) H1740 Diploid cytotype (2*n* = 2*x* = 20). (**B**). H2309B Tetraploid cytotype (2*n* = 4*x* = 40). (**C**) H2319#1 Tetraploid cytotype (2*n* = 4*x* = 40). (**D**) H2674#3 Tetraploid cytotype (2*n* = 4*x* = 40). (**E**) H3010 Diploid cytotype (2*n* = 2*x* = 20). (**F**) H3007#1 Pentaploid cytotype (2*n* = 5*x* = 50). Scale: 10 µm.

**Figure 3:**
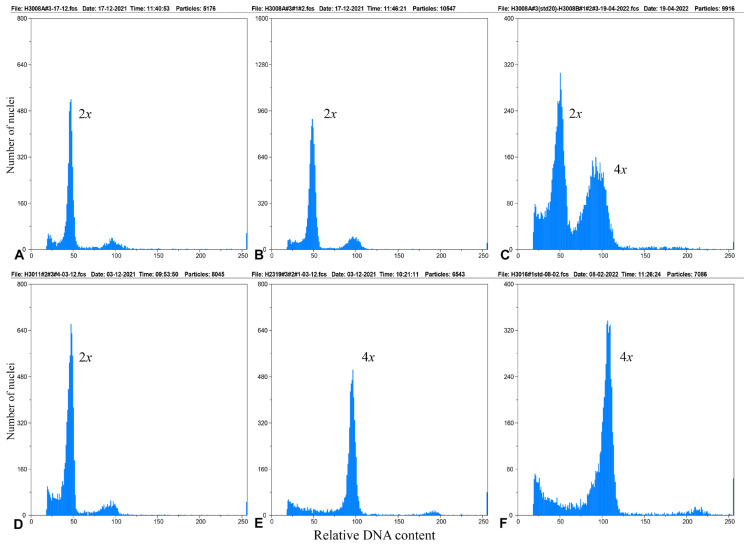
Flow Cytometry Histograms from relative DNA content of cell nuclei of leaves samples showing different ploidy levels in *Paspalum notatum*. (**A**). Histogram of a diploid plant (2*n* = 2*x* = 20) from accession H3008A#3. (**B**). Histogram of a bulk sample from the accesion H3008A#3, #2, #1. All plants are diploids (2*n* = 2*x* = 20). H3008A#3 is the standard plant. (**C**) Histogram of a bulk unknown-ploidy sample from the accesion H3008B#1,#2,#3 and the diploid standard (H3008A#3). Note the tetraploid peak of the sample. (**D**). Histogram of a bulk sample from the accesion H3011#2, #3, #4. All plants are diploids (2*n* = 2*x* = 20). (**E**). Histogram of a bulk sample from the accesion H3008A#3, #2, #1. All plants are tetraploids(2*n* = 4*x* = 40). H2319#1 is the standard plant. (**F**). Histogram for ploidy analysis of H3016#1 accession with 2*n* = 4*x* = 40 chromosomes.

A total of 168 *P. notatum* plants were analysed chromosomally, of which 63 were diploids, 103 tetraploids, and 2 pentaploids. All analysed accessions of *P. notatum* var. *saurae* were diploid (2*n* = 2*x* = 20, Table 1, Figure 1A–D, Figure 2A,E and Figure 3A,B,D). The two populations studied (H1961-Arg and H1740-Py) are monoploids (Figure 1A–C). Individuals from H1961 population were caespitose plants growing uniformly over sandy soils in the river margins (Figure 1A). This population showed strong rhizomes that tolerated flooding (Figure 1B). The H1961 accession was from Gualeguaychú (Argentina) and it was the most south-eastern natural diploid population in Argentina (Table 1, Figure 3A,B).

**Figure 4:**
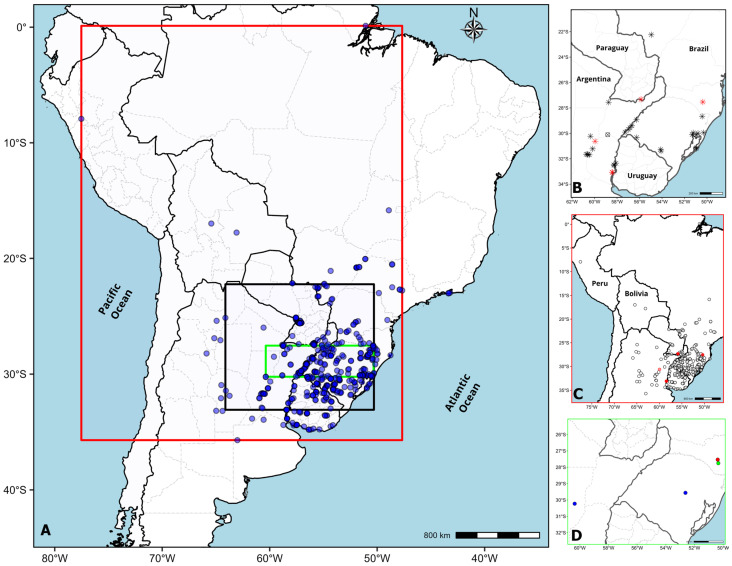
Cytogeography of *Paspalum notatum* cytotypes in South America. The box colours represent the extent of the zoom performed on maps B, C and D. (**A**) Spatial distribution of chromosome counts for *P. notatum.* Blue dots represent the geographic distribution of all localities considered for this study. Note the white areas without dots as gaps in chromosome knowledge about cytotypes living throughout the countries. (**B**) Diploid cytotype geographical distribution. Black asterisk = records of diploid specimens from literature review; red asterisk = records of new diploid specimens contributed by this work; circle and cross = records of triploid specimens from literature review. (**C**) Tetraploid cytotype geographical distribution. White dots black border = records of tetraploid specimens from literature review and new tetraploid specimens contributed by this work; red dots = records of new diploids contributed by this work. (**D**) Pentaploid and hexaploid cytotype geographical distribution. Pentaploid specimen records from literature review = green dot; new record contributed by this work = red dot; hexaploid specimen records from literature review = blue dots.

Here, we reported for the first time an ectopic area for diploid *P. notatum* var. *saurae* in Itapúa, Paraguay (H1740, Table 1, Figure 4A,B). The H1740 population had more delicate individuals, with hard horizontal rhizomes, and trampling tolerance in the surrounding grasslands used such as sports fields.

Our chromosome counts agreed with previous reports for tetraploid bahiagrass (Table S1 and its references). Individuals of *P. notatum* var. *notatum* were consistently tetraploids with one exception (Table 2, Figure 2B–D,F and Figure 3C,E,F). The locality in Santa Catarina near Rio Canoas showed individuals with three ploidy levels (2*x*, 4*x*, 5*x*) (Table 1 and Table 2, Figure 2F, accessions H3007, H3008).

### 3.2. Cytotype Geographical Distribution

**Figure 5:**
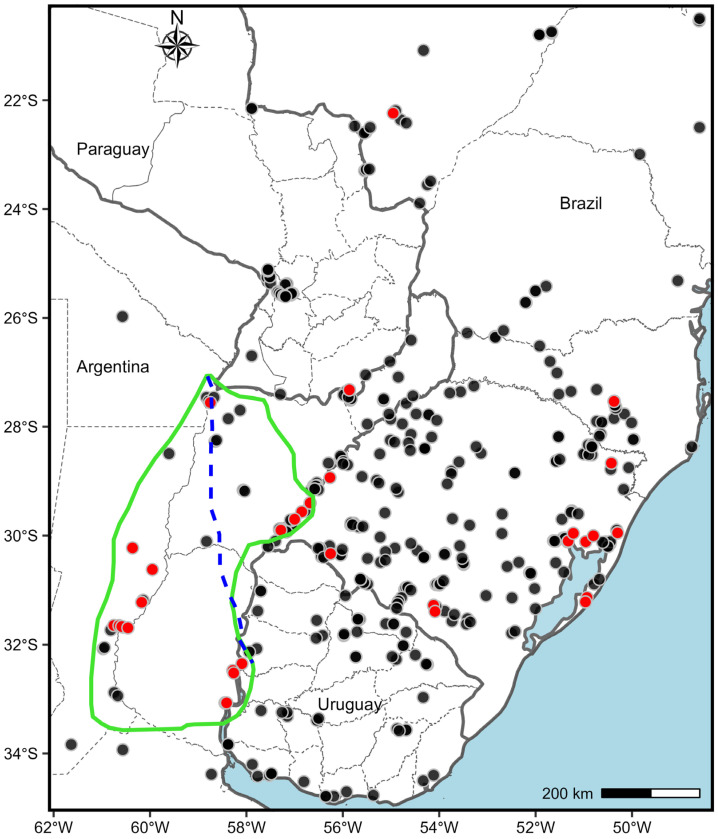
Diploid-tetraploid distribution area of *Paspalum notatum*. Diploid cytotype (red dots) is distributed inside the center of origin of the species (green line) and in ectopic localities in Brazil and Paraguay. Tetraploids (black dots) are positioned around the diploid primary center and extend across a larger area. All ectopic diploids are located outside the region marked with a green line, and are outside of cultivated areas. The dotted blue line indicates the limits of the suggested possible area of origin for the species. All dots represent 2*x* and 4*x* accessions detailed in Table S1.

Collections of the species were made from northeastern Argentina, central Paraguay and as far away as Rio de Janeiro (Brazil), exhibiting the great capacity of bahiagrass to adapt to a wide range of habitats. Within this distribution area, a total of 168 accessions from Argentina, Brazil, and Paraguay were obtained (Table 1 and Table 2). Sixty accessions were identified as *P. notatum var. notatum* tetraploids (2*n* = 4*x* = 40), confirming this cytotype as the most widespread (Table 2). The remaining five accessions were identified as *P. notatum var. saurae* (2*n* = 2*x* = 20), and these localities represent new centers of diversification for Bahiagrass. Accessions H1740, H3008B, H3010 and H3011 are located distant from the center of origin of the species (Table 1, Figure 4A–C and Figure 5). The distance between the center of origin and these neocenters (Table 3) of the diploid cytotype suggests dispersal events and subsequent establishment resulting from the naturalization of diploid cultivars (Figure 4B). These areas can be categorized as hotspots for the conservation of germplasm of diploid bahiagrass.

The chromosome counts available in the literature and in the present work of *P. notatum* are projected onto a South American map (Figure 4A). Cytogenetic survey is concentrated in Argentinian Mesopotamia, south of Brazil, Oriental Region of Paraguay and Uruguay (Figure 4A). Gaps in chromosome analyses of *P. notatum* in South America comprise several countries (Figure 4A, white areas).

Diploids occupy native species areas and ectopic localities outside from the 2*x*- center of origin of the species (Figure 4B). Several diploids escaping from cultivation were also found in Brazil and Paraguay (Figure 4B). Rare and transient triploids were reported for the species in a natural population at Corrientes (Figure 4B). Tetraploids distributed in the biggest area and represented the most frequent cytotype in the natural populations (Figure 4C). Pentaploids were first collected by B. Burson in 1979 (accession USDA PI404863) and in this work (Figure 4D). Unfrequently, hexaploids were reported (Figure 4D), though the possible hexaploid from Brazil is viewed with doubt.

The primary center of origin of the species is in south Entre Rios and Santa Fe up to the north-eastern of Corrientes (Argentina) and extends to Rio Grande do Sul (Brazil) and Paysandú (Uruguay) riverbanks of Uruguay River (Figure 5, Table 3). Outsider diploids were found in Itapúa, Paraguay and Santa Catarina and Rio Grande do Sul, Brazil (Figure 5). Diploids are in close contact with tetraploids in limits of the primary centre of diversity and, also in neonative diploid-polyploid centres of *P. notatum* (Figure 5, Table 3).

## 4. Discussion

### 4.1. Chromosome Numbers and Ploidy Levels in Bahiagrass

*Paspalum notatum* shows several intraspecific ploidy levels, mainly 2*x* and 4*x*, and rare 3*x* and 5*x* [12,48]. Our chromosome counts confirmed that diploid and tetraploid cytotypes are the most common in nature. However, we also identified a pentaploid cytotype in Brazil in a multiploid location where it coexists with 2*x* and 4*x*. The previous report of a solitary pentaploid plant was made by Tischler & Burson [27]. Moreover, both pentaploid accessions are located near each other (Table 1 and Table S1, [27]). Plants with 2*n* = 5*x* = 50 and 2*n* = 6*x* = 60 chromosomes have been obtained experimentally by pollinating apomictic tetraploids plants with pollen from diploid and tetraploid plants, respectively [19,34,49]. Thus, this pentaploid plant could have derived from the natural pollination between tetraploid and diploid plants from the same location.

### 4.2. Primary Gene Pool and Origin Center of P. notatum

The center of origin of *P. notatum* species was probably southern Brazil, Paraguay, northeastern Argentina, and Uruguay [21,22]. This is a frequent and abundant species in the subtropical grasslands of Rio Grande do Sul (Brazil), Mesopotamia (Argentina), and Uruguay [19,21,22], where it is highly variable and serves as an excellent forage and prairie grass, highly resistant to trampling.

The natural occurrence of diploid Bahiagrass is restricted to Argentina—eastern Santa Fe and western Entre Ríos and Corrientes- along the margins and islands of the Paraná River and its tributaries [24]. Moreover, the type specimen of the *saurae* variety, was collected at Concepcion del Uruguay (Entre Rios, Argentina) and was diploid [22,50]. The first record of diploids inhabiting atypical areas in Argentina was described by Quarin [51], who found a diploid accession (Q1740) in Corrientes. More recently, diploid populations and multiploid diploid-tetraploid parapatric and sympatric populations were found at Corrientes by [18]. The current diploid area of *P. notatum* var. *saurae* comprises the region between Cayastá (Santa Fe) and nearby islands at the Parana River, extending to Concepción del Uruguay and Gualeguaychú (Entre Ríos), and a narrow strip along the Parana River up to Corrientes City at north. Our findings in Gualeguaychú (Entre Ríos), confirm the eastern limit of native diploid distribution in Argentina. Cidade et al. [29] observed that the diploid accessions from Uruguaiana and Capivarí do Sul in Rio Grande do Sul, near the Uruguay River, were located in carefully protected areas and may have been transported via the water-flow of the Jacuí and Uruguay Rivers.

Historical germplasm collection of *P. notatum* conserved at the Germplasm Bank of the United States Department of Agriculture (USDA) were recently chromosome counted by [20]. Four new diploids genotypes belonging to seed lots from southern Brazil and Argentina were recovered for breeding programs [20]. An ancient accession found in the USDA germplasm bank (USDA PI404863, 87N) from Paysandú (Uruguay) was diploid [20], which could represent a vestigial locality near the Uruguay River. All these data suggest that the natural 2*x* area includes the riverbanks edges of both margins of the Uruguay River and some of its tributaries.

In summary, our new finding in Entre Ríos (H1961 accession), combined with previous data, supports the hypothesis that this area in Argentina is the origin center of the diploid cytotype. Thus, according to Harlan & de Wet’s [6] classification, the primary gene pool of *P. notatum* could be located in this area.

### 4.3. Neonative Diploid-Polyploid Centers of P. notatum

Cytogenetically, cultivated plants and their wild relatives can share the same ploidy level and can hybridize frequently in nature [52]. If the chromosomes of the hybrids pair perfectly during meiosis, they can originate and establish a fertile population [52]. In species like *P. notatum*, with diploid-polyploid intraspecific polymorphism, cytogeography helps to understand distribution patterns and dispersal dynamics, as polyploids commonly derive from diploids [53]. Harlan [54] used the term areas of varietal diversity to describe secondary diversity areas of crops whose centers of origin are located far away.

Parapatric and sympatric boundaries between diploids and tetraploid natural populations of *P. notatum* were found in Corrientes (Argentina), exhibiting ploidy-dependent habitat preferences [18]. At Riachuelo (Corrientes, Argentina) the authors described a microscale ploidy structure, where diploids were distributed on the inundation bed very close to the stream margins [18]. The Riachuelo population shares the same ecological habitat as the diploid population H1961. In the original natural diploid area like H1961, contiguous tetraploid populations inhabit areas such as terraces with gentle slopes and patchy soils, forming a mosaic transition of 2*x*–4*x* coexistence, which arises from the adaptation of each ploidy to different patchily distributed environment.

Anthropogenic translocations through cultivar escapes mirror the scenario of 2*x–*4*x* natural coexistence. The Pensacola *cv.* of Bahiagrass has been cultivated as forage or turf in many temperate and warm regions of the Americas [34], and from there it can escape into natural grassland areas [17,20,55]. The first report of ectopic diploid accessions of *P. notatum* from South and West Central Brazil was observed by Pozzobon and Valls [15]. In an exhaustive field and cytological study, they reported 11 accessions with 2*n* = 20 among the 127 *P. notatum* accessions and considered the diploid plants to be escapes from the Pensacola cultivar. This ectopic collection of diploid *P. notatum* was likely the result of seed transportation by flooding or river flow from Pensacola *cv.* used as forage in southern Brazil or northern of Rio Grande do Sul (Brazil) [17]. Dahmer [55], also found one diploid accession of *P. notatum* in Candoi (Parana, Brazil, accession V14869), assumed to be a Pensacola *cv.* escape among 93 accessions from Brazil, Argentina and Uruguay. Thus, there are at least two ectopic places for diploid Bahiagrass reported in Rio Grande do Sul and Parana (Brazil) [17,55].

These ectopic places with naturalized diploid *P. notatum* var. *saurae* can be considered neonative centers of diploid Bahiagrass. In these secondary contact zones, diploids and polyploids coexist, interacting through gene flow and intraspecific hybridization. As the tetraploid cytotype of *P. notatum* is the most widespread cytotype, and the diploid natural origin center is a restricted area in Argentina, the identification of neo-diploid centers can be achieved through chromosome counts and/or ploidy determinations using flow cytometry. Phenotypic comparisons using morphological, phenological and ecological markers between natural diploids and naturalized Pensacola *cv.* remain lacking.

The location of these neocenters, distant from the species’ center of origin, suggests dispersal events resulting from the naturalization of diploid cultivars and represents valuable centers of genetic diversification. These neocentres are important hubs of genotype and cytotype diversification. Furthermore, from a conservation perspective, these newly naturalized diploid populations can be categorized as *hot spots* for in situ germplasm preservation. Considering that diploid Bahiagrass is always sexual, these neocentres of diversity are crucial for genetic improvement programs. Sexuality allows the introduction of genetic variability in homo- and heteroploid intraspecific crosses and can be used as a new source of genetic variation for apomictic tetraploid cultivars through artificial polyploidization [11,56].

### 4.4. Neopoliploidization in Neonative Centers of P. notatum

The tetraploid cytotype of bahiagrass is widespread and polymorphic across America [8,31,57,58,59,60,61,62,63,64,65,66,67,68,69,70,71,72]. In Uruguay, tetraploid *P. notatum* is distributed throughout all the country’s grasslands, being dominant and resistant to grazing due to its dense horizontal rhizomes tightly pressed against the ground [57]. In Argentina, except for the origin center of diploid Bahiagrass, the remaining areas of the country are occupied by diverse tetraploids [18,58,59,60,61,62,63,64,65,66,67,68,69,70,71,72,73,74,75,76,77,78,79]. Steiner et al. [80] evaluated *P. notatum* accessions collected in different locations in Brazil, Uruguay, and Argentina and reported large variation in total forage production and leaf yield, and many accessions being superior to *cv.* Pensacola. Thus, outside the origin center, Argentina hosts tetraploids with extensive diversity [58,80].

Neonative centers of diploid *P. notatum* are *foci* of active neopolyploidization. In these centres, neopolyploids can arise through unilateral –triploid bridge- polyploidization [60,61]. In this model, triploids are transient steps that drive the genetic flow among cytotypes, increasing ploidy levels within the agamic complex. Starting from a diploid pool, triploids arise via 2*n* + *n* crosses. If triploids are fertile, and considering the meiotic behaviour with non-equitative chromosomes segregation, they can act as a bridge in a 2*n* + *n* fertilization forming a neotetraploid [81,82]. Thus, autotetraploids may arise in two steps. Triploid cytotypes of *P. notatum* have been occasionally found as individual plants in the limit of 2*x*–4*x* coexistence area in northeastern Argentina, but never as a population [18].

Moreover, ploidy variation can lead to the presence of pentaploid individuals, such as those found in Santa Catarina (H3008), indicating that a third cycle of polyploidization may arise by 2*n* + *n* by intraspecific hybridization between tetraploids and diploids. Areas with cytotype coexistence are optimal scenarios for recurrent reciprocal crosses (4*x* × 2*x*). An unreduced egg cell from a tetraploid plant fertilized by a reduced sperm nucleus from a diploid plant forms a pentaploid ([2*n* = 4*x*] + [*n* = *x*] = 5*x*). Evidence for this polyploidization pathway includes the 5*x P. notatum* found again after 40 years from the first collection made by B. Burson in 1979 (PI508825) [27], in a location near the accession reported here. These pentaploids, at least 40 years old, are undergoing demographic establishment.

The emergence of new odd cytotypes contributes to the cytotype diversity of the agamic complex and may participate in future rounds of polyploidization (if fertile). Neopentaploids constitute a pivot for the next escalation of the polyploidization, both in terms of demographic establishment and participation in new 2*n* + *n* hybridizations. The difference in ploidy levels between polyploid and diploid progenitors acts as a strong reproductive barrier, except when intraspecific intercytotype hybridization occurs producing new polyploids such as B_III_ hybrids, which are ploidy-transgressive relative to their parents [49]. Future studies exploring the reproductive behaviour and genetic system of these neopentaploids could provide further evidence for the unilateral polyploidization described in Bahiagrass.

### 4.5. Neonative Centers and Neighbouring Microscale Dynamics

Agamic complexes comprise sexual and apomictic ecotypes, commonly differing in their ploidy level [83,84]. Moreover, several agamic complexes exhibit geographical parthenogenesis, where apomictic cytotypes are widespread and sexual cytotypes are geographically restricted, *e.g*., *Paspalum intermedium* Munro ex Britton *Ranunculus, Taraxacum* [85,86,87,88]. Natural range expansion may occur through small-scale fluctuations at range margins or via long-distance dispersal events [2].

The geographical distribution of *P. notatum* follows a geographical parthenogenesis pattern, with a native restricted area for sexual diploids and a widespread native area for apomictic tetraploids. However, the introduction and naturalization of Bahiagrass cultivars due to anthropogenic land-use changes (e.g., livestock) have created neocenters of diploid cytotypes distributed separately across the tetraploid area. Diploids in these neocenters have escaped from cultivation, increasing the likelihood of secondary contact zones among ploidy levels (naturalized diploids—native tetraploids).

Several anthropogenic environmental changes have resulted in latitudinal and altitudinal range reductions and expansion [2]. In this context, introductions and escapes of cultivated plants impact local populations both ecologically and genetically [4]. First, contact zones between wild and cultivated species become focal points for genetic interactions such as gene flow and gene introgression. Second, the demographic establishment of ectopic sexual diploid populations of *P. notatum* within native apomictic tetraploids areas creates niche competition among cytotypes, leading to coexistence or displacement. Third, these neopopulations constitute neocenters of ectopic “primary” gene pools -diversity reservoir that could provide new genetic resources for germplasm conservation and breeding programs.

Diploids that escape cultivation and become demographically established act as dispersal kernels toward surrounding areas, serving as recurrent sources of genetic variability. The likelihood of interaction with natural tetraploid *P. notatum* is temporally high. A dispersal kernel defines the likelihood that a seed released from one location, will reach and establish at another. Seeds can be moved by various vectors and may be re-dispersed multiple times before settling [89,90]. Understanding these dispersal kernels is therefore crucial for designing effective in situ conservation strategies. Management plans should consider establishing buffer zones or monitoring gene flow between neonative diploid populations and surrounding tetraploid grasslands to preserve the diploid gene pool integrity or manage the creation of novel polyploids. The spatial shape of the 2*x* neonative dispersal kernel may vary over time and is key to cytotype diversity in the neocenter. An irregular edge increases the perimeter and thus, the potential contact between cytotypes inside and outside the neocenter.

Anthropogenic hybridization is considered a breakdown of reproductive isolation between species due to human actions, such as species introduction, habitat disturbance, or escape of domesticated species [91]. Recurrent introductions of cultivars into native area act as cumulative inputs of genetic and cytotype diversity. In Bahiagrass, the cultivated sexual cytotypes (e.g., *Pensacola* cv.) were introduced as forage in natural grasslands in South America (e.g., south Brazil and west Paraguay), thereby introducing a new cytotype (diploid) into the tetraploid natural distribution. Ultimately, gene flow in contact zones between naturalized diploids and native tetraploids of bahiagrass can originate hybrids with other ploidies, like triploids and pentaploids. Sympatric coexistence of multiple ploidy levels will depend on gene flow intensity and the survival and fitness of the progeny. The expression of apomixis may provide reproductive assurance and facilitate the establishment of new cytotypes.

Biological conservation strategies for these centers require an exhaustive assessment of the cytotype proportion at microscale level. Based on the results presented in this study, germplasm collection strategies need previous ploidy level information to obtain the greatest possible genetic diversity in each population. The design should take into account that in some localities, diploids and polyploids coexist, differing in their reproductive mode. Sexual diploid allogamous *P. notatum*, require a different sampling strategy than tetraploids, which are apomictic pseudogamous and self-compatible. Furthermore, the genetic system of pentaploid *P. notatum* remains unknown. The sampling efforts, distance between plants and population representation and other sampling strategies are different between sexual and apomictic *P. notatum*. Furthermore, the genetic system of pentaploid *P. notatum* remains unknown. However, conserving seeds and living plants ex situ may be insufficient to protect these neocenters. In situ conservation efforts should include the legal protection of natural grasslands against the land-use land-cover changes produced by human activities in South America. For example, Overbeck et al. [92] proposed for Brazilian grassland in situ conservation the creation of new large protected areas, the enforcement of legal restrictions of land use and improvement of monitoring these land-cover changes and the improvement of ecosystem management and sustainable use in governmental programs. Concomitantly, the land use of natural grasslands for livestock production, requires further investigation to better define optimal grazing levels [93], in order to support seed recruitment from both conservation and production perspectives. The widespread public misperception that grassy ecosystems are less valuable or less biodiverse [94] highlights the need for greater educational efforts and cultural change to address the persistent bias that leads to the neglect of these ecosystems globally—despite their exceptionally high biodiversity [94,95]. The neonative centers serve as a source of new inputs of biodiversity conservation.

### 4.6. Further Prospects

Accidental escapes of cultivars into natural populations are expected. Consequently, one can observe in nature anything from the ephemeral coexistence of cultivated and native forms to full invasions [2]. The coexistence of diploid-tetraploid cytotypes in *Paspalum notatum* will depend on the cytotype fitness, reproduction mode, and adaptive tolerance. Diploid *P. notatum* reproduces sexually and forms discrete population, whereas tetraploid bahiagrass reproduces apomictically and forms a *continuum* of local populations across a wider area.

Hybridization poses a challenge for conservation and represents a risk to cytotype coexistence of diploids within tetraploid regions of *P. notatum*. Intraspecific competition will influence territorial and niche occupancy [68]. In the short term, reseeding with diploid seeds may help maintain their current distribution boundaries, although expansion will likely be constrained by competitive pressure from well-adapted surrounding tetraploids. In the medium term, the evolutionary dynamics of these new populations remain open-ended. Future prospecting studies may reveal whether coexistence introduced by diploid migrants within tetraploid zones resembles the pattern observed at native range boundaries.

Conserving diploid *P. notatum* neocentres—as a strategy for safeguarding genetic diversity- can be framed as a set of direct actions integrating mitigation, restoration and reversal of crop and varietal diversity loss. In situ ecological and genetic studies remain entirely unexplored in neocenters like those identified in this work. These neocenters represent active, human-mediated evolutionary experiments. From a conservation perspective, they are invaluable not merely for the static diversity they currently hold, but for the ongoing evolutionary processes they represent. Conserving these sites means protecting the potential for adaptation and the generation of novel genotypes -a critical strategy in the face of climate change and other anthropogenic pressures.

## 5. Conclusions

The new diploid populations of *P. notatum* var. *saurae* are escapes from cultivated *P. notatum*. These escapes have resulted in the naturalization of diploid cultivars, and consequently, they represent new secondary centers of genetic diversity within the diploid gene pool of the species. These neocenters have been established within the native area of tetraploid bahiagrass, thus creating new contact zones among cytotypes. These mixed-ploidy neocentres of bahiagrass are *foci* of recurrent neopolyploidization through intraspecific hybridizations. These neonative centres could enrich germplasm sources of Bahiagrass, and therefore, are crucial areas for in situ conservation.

This study fundamentally redefines the cytogeographic landscape of *Paspalum notatum* by identifying novel, human-influenced neocentres of diploid-polyploid diversity. These are not mere geographical curiosities but dynamic evolutionary arenas that are critical for biodiversity conservation. We demonstrate that the escape of cultivated plants is actively generating new *genetic hotspots*, providing essential raw material for both crop improvement and ecological resilience. Our findings underscore the urgent need to incorporate these neonative centres into germplasm collection and in situ conservation policies, shifting the focus from preserving static genetic relics to managing the ongoing evolutionary processes that are vital for adaptation in a changing world. This work provides a new, actionable framework for biological conservation in anthropogenic landscapes.

## Figures and Tables

**Table 1:** List of the studied *P. notatum* var. *saurae* accessions, their collection number (ID), the number of plants analyzed (N), ploidy level (*x*) and their provenance.

x	ID (N)	Provenance
2*x*	H1961 #3 to #29 (27)	Argentine. Entre Ríos, Gualeguaychú.
2*x*	Q 4119 (1)	Argentine. Santa Fe, 7 km S from San Javier, (CTES Herbarium, Quarin unpubl.)
2*x*	H1740 #1, #3 to #27 (26)	Paraguay. Itapúa, Encarnación.
2*x*	H3008 #1 to #4 (4)	Brazil. Santa Catarina, BR-116, 5 km N Rio Canoas. [*ca*. V8211]
2*x*	H3010 #1, #2 (2)	Brazil. Santa Catarina, BR-116, 5 km N Rio Canoas. [*ca*. V8211]
2*x*	H3011 #2 to #4 (3)	Brazil. Santa Catarina, BR-116, 5 km N Rio Canoas. [*ca*. V8211]

Collectors: H, Honfi A. I.; Q, Quarin C.L. Countries: A, Argentine, B, Brazil, P, Paraguay. Diploids, 2*n* = 2*x* = 20.

**Table 2:** List of the studied *P. notatum* var. *notatum* accessions, their collection number (ID), the number of plants analyzed (N), ploidy level (*x*) and their provenance.

x	ID (N)	Provenance
4*x*	H1271 (1)	Argentine. Misiones, Guaraní, Arroyo Yerbas del Paraiso.
4*x*	H1350 (1)	Argentine. Misiones, Capital, Bañados del Zaimán.
4*x*	H2137 (1)	Argentine. Corrientes. Paso de los Libres city. Bartolomé Mitre Square.
4*x*	H2543 #1 to #3 (3)	Argentine. Misiones, Eldorado, Provintial Route 17, 11 km near Pozo Azul.
4*x*	H2554 #1 to #5 (5)	Argentine. Misiones, Capital, Garupá, near the Train Station.
4*x*	H2665 (1)	Argentine. Misiones, Concepción de la Sierra, Provintial Routea 2.
4*x*	H2671 (1)	Argentine. Misiones, Ruta provincial 2, El Palmar del Río.
4*x*	H2674 #1 to #11 (11)	Argentine. Misiones, Oberá, Panambí, Ruta provincial 2.
4*x*	H2295 #1 to #3 (3)	Brazil. Parana, Route BR-280, A 57 km from Francisco Beltrão.
4*x*	H2304 #1,#2,#6,#7 (4)	Brazil. Santa Catarina, BR-116. 1,5 km S da prefeitura of Correia Pinto. [ca. V14411].
4*x*	H2309B (1)	Brazil. Santa Catarina, SC-114 de Urupema a Lages, near Lages. [ca. V4508, V12156].
4*x*	H2310 #1,#4 (2)	Brazil. Santa Catarina, Correia Pinto, BR-116 y Rua Duque de Caxias y Rua Cándido [ca. V14411].
4*x*	H2312 #1 to #5 (5)	Brazil. Santa Catarina, SC-114 de Urupema a Lages, near Lages, [ca. V4508, V12156].
4*x*	H2319 #1 (1)	Brazil. Santa Catarina, BR-116 a 50 km al N de Vacaría [ca. V12159].
4*x*	H2690 (1)	Brazil. Rio de Janeiro, Jardim Botânico do Rio de Janeiro. [H2690RB #8]
4*x*	H2691 #2, #4, #6, #8, #9 (5)	Brazil. Rio de Janeiro, Barra de Tijuca, on the sidewalk.
4*x*	H2693 #1,#3 (2)	Brazil. Rio de Janeiro, Jardim Botânico do Rio de Janeiro.
4*x*	H3003 #1,#3 (2)	Brazil. Santa Catarina, BR-116 km 210.
4*x*	H3007 #2 (1)	Brazil. Santa Catarina, BR-116, 5 km N Rio Canoas. [ca. V8211]
4*x*	H3008 *C* #2, #3 (2)	Brazil. Santa Catarina, BR-116, 5 km N Rio Canoas. [ca. V8211]
4*x*	H3016 #1, #2 (2)	Brazil. Santa Catarina, BR-116 Correia Pinto. [ca. V14411]
4*x*	H2528 (1)	Paraguay. Central. National route 2, a 18 km of Caacupé City.
4*x*	Ch–1 to Ch–3 (3)	Paraguay. Cordillera. Compañía Capilla cué.
4*x*	Ch–4 to Ch–6 (3)	Paraguay. Cordillera. Compañía Capilla cué, on the side of the road Faustino Rivas.
4*x*	Ch–7 to Ch–14 (8)	Paraguay. Cordillera. Compañía Capilla cué.
4*x*	Ch–25 (1)	Paraguay. Central. Luque City, on the side of the road
4*x*	Ch–26 to Ch–27 (2)	Paraguay. Central. Luque, Central promenade of the Ñú Guazú highway
4*x*	Ch–29 (1)	Paraguay. Central. Luque city, on the side of the road
4*x*	Ch–31, Ch–33, Ch 33B to Ch–37 (7)	Paraguay. Paraguarí. On the side of the National route PY1.
4*x*	Ch–38, Ch–40 (2)	Paraguay. Paraguarí. National route PY1, on the roadside, in front of the Yaguarón Hill.
4*x*	Ch–41, Ch–44, Ch–45 (3)	Paraguay. Paraguarí. National route PY1, Paraguarí City.
4*x*	Ch–42 (1)	Paraguay. Paraguarí. National route PY1, on the roadside to access to Paraguarí City.
4*x*	Ch–43 (1)	Paraguay. Paraguarí. National route PY1, on the roadside to Paraguarí City.
4*x*	Ch–47 to Ch–61 (15)	Paraguay. Presidente Hayes. Transchaco route, on the roadside.
5*x*	H3007 #1, #3 (2)	Brazil. Santa Catarina, BR-116, 5 km N Rio Canoas. [ca. V8211]

Collectors: H, Honfi A. I.; Ch, Chaparro C.; V, Valls J.F.M. Tetraploids, 2*n* = 4*x* = 40; Pentaploid 2*n* = 5*x* = 50.

**Table 3:** Summary of several concepts related to genetics of *P. notatum* geographical distribution.

**Glossary**
Neonative: A species or biotype or cytotype which result in a by-product of hybridization between a native species and an alien taxon.
Neonative center: Geographical area of origin of neonative taxa.
Gene pool: Sum of all the genes, alleles, and karyotypes shared by individuals of a species.
Center of origin: Geographical area where the species originated. In species with several intraspecific ploidy levels, the diploid location is an indicator of the evolutionary ancestry starting point for polyploids. In allopolyploid species, the center of origin refers to the site where progenitor species have probably hybridized.
Neocenters: New centers of diversity of a species.
Diversity center: Geographical area with prominent genetic diversity of a species.
Primary center of origin: primordial area with highest morphological and genetic diversity and where is most plausible originated the species.
Secondary diversity center: Geographical area where there is great genetic variability of a crop or species, but which is not the main center of origin of that species.
Ectopic area: Outside area from the natural geographical distribution of the taxon, biotype, cytotype or species.

## Data Availability

Data are contained within the article.

## Supplementary Materials

The following supporting information can be downloaded at: https://www.mdpi.com/article/10.3390/genes16091098/s1, Table S1: Complete list of chromosome data previously reported for *P. notatum.* Figure S1: Methodological Flowchart.

## Abbreviations

The following abbreviations are used in this manuscript:

2n.	Sporophitic chromosome number
*x*	Basic chromosome number, ploidy
*n*	Gametic chromosome number
B_III_	2*n* + *n* hybrid
