# Supplementary material for: Neonative Diploid-Polyploid Hotspots of Paspalum notatum: Identifying Novel Genetic Diversity for Conservation in South America
Source: Genes (Basel). 2025 Sep 16;16(9):1098. doi: 10.3390/genes16091098 (PMC12470053; doi:10.3390/genes16091098)
Supplement: Supplementary file 1 [file genes-16-01098-s001.zip › genes-3780646 manuscript revised Table S1 VF.pdf]

Table S1. Data base of chromosome numbers of *Paspalum notatum* (*x*=10)

Reference	*x*	Specimen	Provenance
[1]	2*x*	S/D	ARG, Entre Ríos, Concepción Del Uruguay
[2]	2*x*	PI 149303	ARG, Santa Fe
[3]	2*x*	Q 1750	ARG, Corrientes, Puente Pexoa, riverside
[4,5,6,7]	2*x*	Q 4084	ARG, Santa Fe, Cayastá
[8]	2*x*	Q 4060 (ARG-23)	ARG, Salta, 10 km S of J.V. Gonzalez.
[5]	2*x*	Q 4175 (ARG13)	ARG, Santa Fe, 15 km W of La Criolla.
[9]	2*x*	R2X	ARG, Corrientes, Riachuelo Stream, 15 Km SE Corrientes City
[10]	2*x*	67N PI337573	ARG, Santa Fe, Berduc Island, in Parana river, east of Santa Fe
[10]	2*x*	92N PI 508831	ARG, Santa Fe. Isla El Timbó, W side of Parana River between Parana and Santa Fe.
[10]	2*x*	92N PI 508832	ARG, Santa Fe. Isla El Timbó, W side of Parana River between Parana and Santa Fe.
[11]	2*x*	H1453 # A	ARG, Santa Fe, Ruta provincial 19, Colastiné, near to Tunel subfluvial.
[12]	2*x*	H1453#1, #2	ARG, Santa Fe, Ruta provincial 19, Colastiné, near to Tunel subfluvial.
[12]	2*x*	H1961#3, #11	ARG, Entre Ríos, Gualeguaychú
[13]	2*x*	PI 508836	ARG, Entre Rios, Near Rio Uruguay at Concepción del Uruguay
[13]	2*x*	PI 508848	ARG, Santa Fe, Isla Santa Candida, near W bank of Rio Paraná between Parana and Santa Fe
[13]	2*x*	PI 291-79	ARG, Santa Fe, Isla Santa Candida, near W bank of Rio Paraná between Paraná and Santa Fe

[14]	2*x*	#R1 (voucher CTES0553130)	ARG, Santa Fe, Cayastá
[15]	2*x*	BRA-12351, V 10036	BRA, RS, Mostardas
[15]	2*x*	BRA-17205, V 12342	BRA, RS, Dom Pedrito
[15]	2*x*	BRA-17361, V 12370	BRA, RS, Santana do Livramento
[15]	2*x*	BRA-17507, V 12391	BRA, RS, Uruguaiana
[15]	2*x*	BRA-17647, V 12409	BRA, RS, Uruguaiana
[15]	2*x*	BRA-17680, V 12414	BRA, RS, Uruguaiana
[15]	2*x*	BRA-17701, V 12416	BRA, RS, Uruguaiana
[15]	2*x*	BRA-18627, V 12830	BRA, RS, Uruguaiana
[15]	2*x*	BRA-7641, V 4067	BRA, RS, Bom Jesus
[15]	2*x*	BRA-7803, V 4669	BRA, RS, Itaquí
[15]	2*x*	BRA-7901, V 4768	BRA, RS, Osório
[16,17]	2*x*	BRA-006173	BRA, RS, Bagé
[18]	2*x*	M02 M Dallagnol s/n	BRA, RS, Viamao
[18]	2*x*	M03 M Dallagnol s/n	BRA, RS, Viamao
[18]	2*x*	M04 M Dallagnol s/n	BRA, RS, Viamao
[18]	2*x*	M05 M Dallagnol s/n	BRA, RS, Viamao
[18]	2*x*	M06 M Dallagnol s/n	BRA, RS, Viamao
[18]	2*x*	M09 M Dallagnol s/n	BRA, RS, Viamao

[18]	2*x*	M10 M Dallagnol s/n	BRA, RS, Viamao
[18]	2*x*	MD S/N	BRA, RS, Viamao
[19,20,21])	2*x*	PI 310163	BRA, RS, Guaiba.
[22-24]	2*x*	Rua 296	BRA, Mato Grosso do Sul, Dourados.
[25]	2*x*	St s/n	BRA, RS, Viamão
[18,25]	2*x*	V 14244 ''A''	BRA, RS, Uruguaiana
[18,25]	2*x*	V 14829, BRA - 024236	BRA, RS, Candói Rodovia BR 277
[25]	2*x*	V 9607, BRA - 006173	BRA, RS, Bagé
[10]	2*x*	87N PI404863	URU, Paysandú. On Route 26, 109 km east of Paysandú.
[12]	2*x*	H1740#2	PY, Itapúa, Encarnación.
This work	2*x*	Q 4119	ARG, Santa Fe, 7 km S de San Javier CTES Herbarium (Quarin Unpubl.)
This work	2*x*	H1961#3-#29	ARG, Entre Ríos, Gualeguaychú
This work	2*x*	H1740#1, #3-#27	PY, Itapúa, Encarnación.
This work	2*x*	H3008B	BRA, Santa Catarina, BR116, 5 km N Rio Canoas.
This work	2*x*	H3010	BRA, Santa Catarina, BR116, 5 km N Rio Canoas.
This work	2*x*	H3011	BRA, Santa Catarina, BR116, 5 km N Rio Canoas.
[26,8-9]	3*x*	Q3686	ARG, Corrientes,18 km N of Sauce, Paso Mula.
[27]	3*x*	GOULD 11658	MX, Oaxaca, 92 Miles South of Oaxaca
[13]	3*x*	S/D	USA, FLORIDA, 6 Km E Bradenton

Paspalum notatum var. *notatum*			
[28]	4*x*	C-16/24	ARG, Córdoba, Córdoba
[28]	4*x*	LC-1	ARG, Córdoba, Los Cocos
[28]	4*x*	Ma-6	ARG, Corrientes, Estero de Las Maloyas, Route 5
[5]	4*x*	Obera	ARG, Misiones, Oberá
[28]	4*x*	Q0845	ARG, Santa Fe, Villa Ana
[28]	4*x*	Q3774	ARG, Misiones, Puerto Rico
[5,28]	4*x*	Q3778	ARG, Corrientes, Palmar Grande between Caá Cati and Mburucuyá
[28]	4*x*	Q3783	ARG, Buenos Aires, Pergamino
[4,28]	4*x*	Q3838	ARG, Corrientes, 15 KM S Corrientes City
[5,29-31]	4*x*	Q3838, ARG-04	ARG, Corrientes, Riachuelo stream and Route 12.
[5,28]	4*x*	Q3845, ARG-05	ARG, Corrientes, 18 km N of Sauce, Paso Mula
[5]	4*x*	Q3931	ARG, Corrientes, Saladas.
[5,30-31]	4*x*	Q4064	ARG, Corrientes, Saladas
[5]	4*x*	Q4210, ARG-17	ARG, Catamarca, 10 km of Catamarca on the road to El Rodeo.
[5]	4*x*	Q4261, ARG-18	ARG, Corrientes, Santa Ana, male-sterile plant
[5,31]	4*x*	Q4270	ARG, Santa Fe, Rosario, Patio de la Madera.
[9]	4*x*	C4X	ARG, Chaco, 11,5 KM SE from Castelli City
[10,29]	4*x*	30N PI508833	ARG, Santa Fe, Route 11, 35km S of Santa Fe.

[10,29]	4*x*	36N PI 508834	ARG, Santa Fé, Route 11, Arocena, 67km S of Santa Fe.
[10,29]	4*x*	48N PI 508838	ARG, Corrientes, Mercedes, At Corrientes River bridge on Route 23 W of Mercedes.
[10,29]	4*x*	51N PI 337566	ARG, Santa Fé, Cayasta, Santa Fe Province. Along Route 168A.
[10,29]	4*x*	70N PI 508828	ARG, Cordoba, Cordoba Route 9, 1 km W of Manfredi, Cordoba Province.
[10,29]	4*x*	79N PI 508840	ARG, Corrientes Arroyo Cuay, Route 129. .
[10,29]	4*x*	83N PI 508827	ARG, Santa Fe, Grazed area, Route 8, 89km W of Venado Tuerto.
[10,29]	4*x*	95N PI 424652	ARG, Corrientes. Near highway bridge over Rio Riachuelo.
[5]	4*x*	ARG-01 Obera´	ARG, Misiones, Oberá
[5]	4*x*	ARG-20 U46	ARG, Córdoba, Río Cuarto.
[5]	4*x*	ARG-21 U47	ARG, Corrientes, Parque Mitre, male-sterile plant.
[5]	4*x*	ARG-22 U48	ARG, Corrientes, Mercedes.
[25]	4*x*	CN s/n	ARG, Corrientes, Santo Tome
[25]	4*x*	CN s/n	ARG, Corrientes, Santo Tome
[25]	4*x*	CN s/n	ARG, Corrientes, Santo Tome
[25]	4*x*	CN s/n	ARG, Corrientes, Santo Tome
[25]	4*x*	CN s/n	ARG, Corrientes, Santo Tome
[25]	4*x*	CN s/n	ARG, Corrientes, Santo Tome
[25]	4*x*	CN s/n	ARG, MISIONES, Posadas
[25]	4*x*	CN s/n	ARG, Corrientes, Santo Tome

[25]	4*x*	CN s/n	ARG, Corrientes, Santo Tome
[25]	4*x*	CN s/n	ARG, Corrientes, Santo Tome
[25]	4*x*	CN s/n	ARG, Corrientes, Santo Tome
[25]	4*x*	CN s/n	ARG, Misiones, Posadas
[25]	4*x*	CN s/n *	ARG, Corrientes, San Tome´
[30]	4*x*	Cy A1556	ARG, Buenos Aires, Km. 364 near Fortín Olavarría
[11-12]	4*x*	H1304	ARG, Santa Fe, Santa Fe, surroundings access to the subfluvial tunnel
[11-12]	4*x*	H1603#1	ARG, Misiones, Capital, Posadas.
[11-12]	4*x*	H1603#2	ARG, Misiones, Capital, Posadas.
[11-12]	4*x*	H1603#3	ARG, Misiones, Capital, Posadas.
[11]	4*x*	H220	ARG, Misiones, Capital, Santa Inés,
[22]	4*x*	Hojsgaard 327	ARG, Corrientes, Paso de los Libres, La Cruz.
[18]	4*x*	M36	ARG, Corrientes, Aguapeí (Arapeí) Santo Tome
[18]	4*x*	M37	ARG, Corrientes, Aguapeí (Arapeí) Santo Tome
[18]	4*x*	M38	ARG, Corrientes, Aguapeí (Arapeí) Santo Tome
[18]	4*x*	M39	ARG, Corrientes, Aguapeí (Arapeí) Santo Tome
[18]	4*x*	M41	ARG, Corrientes, Aguapeí (Arapeí) Santo Tome
[18]	4*x*	M42	ARG, Corrientes, Aguapeí (Arapeí) Santo Tome
[18]	4*x*	M43	ARG, Corrientes, Aguapeí (Arapeí) Santo Tome

[18]	4*x*	M44	ARG, Corrientes, Aguapeí (Arapeí) Santo Tome
[18]	4*x*	M46	ARG, Corrientes, Aguapeí (Arapeí) Santo Tome
[18]	4*x*	M51	ARG, Misiones, Posadas
[13]	4*x*	PI 508834	ARG, Santa Fe, ArocenaRoute 11, Arocena, 67km S of Santa Fe
[13]	4*x*	PI 330-79	ARG, Buenos Aires, INgr. Maschwitz (50 Km NW Buenos Aires)
[13]	4*x*	PI 508839	ARG, Misiones, Salto Encantado, 42 Km E of intersection of Routes 12 and route 220
[13]	4*x*	PI 508841	ARG, Tucumán, Route 9, 87 Km NW Santiago del Estero
[13]	4*x*	PI 508842	ARG, Salta, Route 34, 33 Km N Metan
[31]	4*x*	Q4294	ARG, CORDOBA, Los Algarrobos, Santa Rosa de Calamuchita
[9]	4*x*	R4X	ARG, Corrientes, Riachuelo Stream, 15 km SE Corrientes City
[1]	4*x*	S/D	ARG, Córdoba, Achiras
[1]	4*x*	S/D	ARG, Tucumán
[29]	4*x*	V51 MDS/N	ARG, Misiones, Posadas
[5,28,30-31]	4*x*	Q3776	BO, Chapare Region, Villa Tunari
[5,31]	4*x*	ST2369	BO, Santa Cruz de la Sierra, 2km S of Salinas
[18]	4*x*	100 MD & CN s/n	BRA, RS, Rosário do Sul
[18]	4*x*	74 MD & CN s/n	BRA, RS, Tapes
[18]	4*x*	90 MD & CN s/n	BRA, RS, Livramento

[18]	4*x*	96 MD & CN s/n	BRA, RS, Alegrete
[18]	4*x*	MD & CN s/n	BRA, RS, São Gabriel
[18]	4*x*	101 MD& CN s/n	BRA, RS, São Gabriel
[18]	4*x*	105 M DallagnoD & C Nabinger s/n -	BRA, RS, Vila Nova do Sul
[18]	4*x*	106 MD& CN s/n	BRA, RS, Vila Nova do Sul
[10,29]	4*x*	13N PI 404473	BRA, RS, Vacaria, On Route BR 285, 10 km west of Vacaria.
[10,29]	4*x*	20N PI508826	BRA, RS, Guaíba Agronomy Experimental Farm, Guaiba, Rio Grande do Sul. remarks: Plants bunch type, Capivari form. Leaves fine, narrow, 20-30cm long. Inflorescences 45cm high. Wild. Seed.
[10,29	4*x*	33N PI 310169	BRA, RS, Guaíba
[10,29	4*x*	49N PI 310168	BRA, RS, Guaíba
[18]	4*x*	673 MD& CN s/n	BRA, RS, Eldorado do Sul
[18]	4*x*	70 ND s/n	BRA, RS, Porto Lucena
[18]	4*x*	71 ND s/n	BRA, RS, Três de Maio
[10,29]	4*x*	71N PI 310145	BRA, RS, Montenegro
[18]	4*x*	72 ND s/n	BRA, RS, Três de Maio
[18]	4*x*	73 ND s/n	BRA, RS, Cruz Alta
[10,29]	4*x*	73N PI 241878 4x	BRA, SP, Nova Ovinhos, near Ourinhos
[18]	4*x*	75 MD & CN s/n	BRA, RS, Cristal
[18]	4*x*	76 MD & CN s/n	BRA, RS, São Lourenço do Sul

[18]	4*x*	77 MD & CN s/n	BRA, RS, Capão do Leão
[18]	4*x*	78 MD & CN s/n	BRA, RS, Capão do Leão
[18]	4*x*	79 MD & CN s/n	BRA, RS, Pinheiro Machado
[18]	4*x*	80 MD & CN s/n	BRA, RS, Pinheiro Machado
[10,29]	4*x*	80N PI 310170	BRA, RS, Guaíba
[18]	4*x*	81 MD & CN s/n	BRA, RS, Pinheiro Machado
[18]	4*x*	82 MD & CN s/n	BRA, RS, Candiota
[18]	4*x*	83 MD & CN s/n	BRA, RS, Hulha Negra
[18]	4*x*	84 MD & CN s/n	BRA, RS, Bexigoso
[18]	4*x*	85 MD & CN s/n	BRA, RS, Dom Pedrito
[18]	4*x*	86 MD & CN s/n	BRA, RS, Dom Pedrito
[18]	4*x*	87 MD & CN s/n	BRA, RS, Dom Pedrito
[18]	4*x*	88 MD & CN s/n	BRA, RS, Livramento
[18]	4*x*	89 MD & CN s/n	BRA, RS, Livramento
[10,29]	4*x*	93N PI 404476	BRA, RS, Bom Jesus, route to Sao Francisco, 33 km south of Bom Jesus.
[18]	4*x*	95 MD & CN s/n	BRA, RS Alegrete
[18]	4*x*	97 MD & CN s/n	BRA, RS, Alegrete
[18]	4*x*	98 MD & CN s/n	BRA, RS, Alegrete
[30]	4*x*	B229	BRA, RS, Itaquí

[15]	4x	BRA-006301	BRA, RS, QUARAI
[16]	4x	BRA-007986	BRA, RS, Guaíba.
[32]	4x	BRA-008028	BRA, SC, LAGES
[5]	4x	BRA-01 IB229	BRA, RS, Itaquı´
[5]	4x	BRA-02 Q3844	BRA, Lagoa Vermelha, RS.
[25]	4x	BRA-021563	BRA, RS, Capivari do Sul
[5]	4x	BRA-03 Q4008	BRA, Aparecida do Taboado, MS.
[5]	4x	BRA-04 Q4010	BRA, Tres Lagoas, MS
[5]	4x	BRA-05 Q4011	BRA, Tres Lagoas, MS
[5]	4x	BRA-06 Q4012	BRA, Tres Lagoas, MS
[5]	4x	BRA-07 Q4016	BRA, 18 km S of Dourados, MS.
[5]	4x	BRA-08 Q4022	BRA, 30 km E of Ponta Pora, MS.
[5]	4x	BRA-09 Q4023	BRA, 30 km E of Ponta Pora, MS.
[5]	4x	BRA-10 Q4029	BRA, Coronel Sapucaia, MS.
[8]	4x	BRA-12 Q4226	BRA, Restinga Seca, RS.
[25]	4x	CIDADE 003	BRA, RS, Palmitinho
[25]	4x	CIDADE 004	BRA, RS, Tenente Portela
[25]	4x	CIDADE 006	BRA, RS, Santo Angelo
[25]	4x	CIDADE 010	BRA, RS, Tuparendi

[25]	$4x$	CIDADE 012	BRA, RS, Porto Mauá
[25]	$4x$	CIDADE 016	BRA, RS, Candido Godoí
[25]	$4x$	CIDADE 018	BRA, RS, Sao Luiz Gonzaga
[25]	$4x$	CIDADE 022	BRA, RS, Caibaté
[25]	$4x$	CIDADE 023	BRA, RS, Sao Luiz Gonzaga
[25]	$4x$	CIDADE 026	BRA, RS, Sao Francisco de Assis
[25]	$4x$	CN s/n	BRA, RS, André da Rocha
[25]	$4x$	CN s/n	BRA, RS, Eldorado do Sul
[25]	$4x$	CN s/n	BRA, SP, Piracicaba
[25]	$4x$	CN s/n	BRA, SP, Piracicaba
[15]	$4x$	DALLAGNOL 316, BRA-11754	BRA, SC, Capinzal
[15]	$4x$	DALLAGNOL 263, BRA-11657	BRA, SC, Campo Belo do Sul
[15]	$4x$	DALLAGNOL 275, BRA-19232	BRA, SC, Campo Belo do Sul
[15]	$4x$	DALLAGNOL 302, BRA-11703	BRA, SC, Campo Belo do Sul
[25]	$4x$	EEA 673, BRA - 007986	BRA, RS, Eldorado do Sul
[15]	$4x$	GONÇALVES 6, BRA-12301	BRA, RS, Dom Pedrito
[15]	$4x$	GONÇALVES 1, BRA-12271	BRA, RS, Dom Pedrito
[15]	$4x$	GONÇALVES 5, BRA-12297	BRA, RS, Dom Pedrito

[15]	4*x*	GONÇALVES 9, BRA-12335	BRA, RS, Dom Pedrito
[5,28,31]	4*x*	IB 229	BRA, RS, ITAQUI
[18]	4*x*	M23 MD S/N	BRA, RS, Eldorado do Sul
[18]	4*x*	M24	BRA, RS, Eldorado do Sul
[18]	4*x*	M26	BRA, RS, Caçapava do Sul
[18]	4*x*	M27	BRA, RS, Lavras do Sul
[18]	4*x*	M29	BRA, RS, Lavras do Sul
[18]	4*x*	M30	BRA, RS, Bagé
[18]	4*x*	M31	BRA, RS, André da Rocha
[18]	4*x*	M32	BRA, SP, Barretos
[18]	4*x*	M33	BRA, RS, Vacaria
[18]	4*x*	M34	BRA, RS, Vacaria
[18]	4*x*	M35	BRA, RS, São Borja
[18]	4*x*	M49	BRA, RS, Mostardas
[18]	4*x*	M50	BRA, SP, Piracicaba
[18]	4*x*	M52 Steiner s/n	BRA, RS, Santiago
[18]	4*x*	M53	BRA, SC, Agua Doce
[18]	4*x*	M54	BRA, RS, Eldorado do Sul
[18]	4*x*	M69	BRA, RS, São José do Hortêncio

[25]	4x	MD s/n	BRA, RS Cacapava do Sul
[25]	4x	MD s/n	BRA, RS, André da Rocha
[25,33]	4x	MD s/n	BRA, RS, Bagé
[25]	4x	MD s/n	BRA, RS, Caçapava do Sul
[25]	4x	MD s/n	BRA, RS, Caçapava do Sul
[25]	4x	MD s/n	BRA, RS, Encruzilhada do Sul
[25]	4x	MD s/n	BRA, RS, Lavras do Sul
[25]	4x	MD s/n	BRA, RS, Lavras do Sul
[25]	4x	MD s/n	BRA, RS, Lavras do Sul
[25]	4x	MD s/n	BRA, RS, Mostardas
[25]	4x	MD s/n	BRA, RS, São Borja
[25]	4x	MD s/n	BRA, RS, Vacaria
[25]	4x	MD s/n	BRA, SP, Barretos
[25]	4x	MD s/n	BRA, SP, Barretos
[33]	4x	MD s/n	BRA, RS, Cacapava do Sul
[33]	4x	MD s/n	BRA, RS, Lavras do Sul
[33]	4x	MD s/n	BRA, RS, Lavras do Sul
[33]	4x	MD s/n	BRA, RS, Lavras do Sul
[33]	4x	MD s/n	BRA, RS, Sao Borja

[33]	4*x*	MD s/n	BRA, RS, Sao Jose´ do Hortencio
[33]	4*x*	MD s/n	BRA, RS, Vacaria
[18]	4*x*	MD25	BRA, RS, Lavras do Sul
[13]	4*x*	PI 404472	BRA, RS, 5 Km NW Vacaria Agricultural Experiment Station, Vacaria.
[13]	4*x*	PI 404478	BRA, RS, Route BR 293, 44 Km E Bagé. On Route BR 293, 44 km east of Bage.
[13]	4*x*	PI 404481	BRA, RS, Route BR 290, 30 Km E Alegrete On Route BR 290, 30 km east of Alegrate.
[13]	4*x*	PI 404482	BRA, RS, 30 Km W Guaiba. Agricultural Experimental Station, Guaiba, 49 km west of Porto Alegre.
[5,28]	4*x*	Q3844	BRA, RS, Lagoa Vermelha
[28]	4*x*	Q3853	BRA, RS, Osorio-Capivari
[5,34]	4*x*	Q4008, V 11733	BRA, MS, 14 Km Sw de Aparecida Do Taboado
[5,2,31]	4*x*	Q4010	BRA, MS, Tres Lagoas
[5,34]	4*x*	Q4011, V 11882	BRA, MS, 30 Km E De Ponta Pora
[34]	4*x*	Q4012, V 11743	BRA, MS, Tres Lagoas
[5,28]	4*x*	Q4016	BRA, MS, 18 KM S DOURADOS
[5]	4*x*	Q4022	BRA, MS, 30 km E of Ponta Porá
[5,28,31]	4*x*	Q4023	BRA, MS, 30 Km E Ponta Porá
[5]	4*x*	Q4029	BRA, MS, Coronel Sapucaia
[33]	4*x*	SOUZA CHIES 1	BRA, RS, Camaquá

[33]	$4x$	Souza Chies 154	BRA, RS, Cambara´ do Sul
[33]	$4x$	Souza Chies 18	BRA, RS, Dom Feliciano
[33]	$4x$	Souza Chies 193	BRA, RS, Sao Jose´ do Ausentes
[33]	$4x$	Souza Chies 206	BRA, RS, Vacaria
[33]	$4x$	Souza Chies 224 4	BRA, RS, Rosa´rio do Sul
[33]	$4x$	Souza Chies 226	BRA, RS, Rosa´rio do Sul
[33]	$4x$	Souza Chies 238	BRA, RS, Santana do Livramento
[33]	$4x$	Souza Chies 242	BRA, RS, Santana do Livramento
[33]	$4x$	Souza Chies 242	BRA, RS, Santana do Livramento
[33]	$4x$	Souza Chies 244	BRA, RS, Quaraı´
[33]	$4x$	Souza Chies 247	BRA, RS, Quaraı´
[33]	$4x$	Souza Chies 268	BRA, RS, Alegrete
[33]	$4x$	Souza Chies 27	BRA, RS, Dom Feliciano
[33]	$4x$	Souza Chies 271	BRA, RS, Rosario do Sul
[33]	$4x$	Souza Chies 30	BRA, RS, Dom Feliciano
[33]	$4x$	Souza Chies 48	BRA, RS, Encruzilhada do Sul
[33]	$4x$	Souza Chies 83	BRA, RS, Cangucu
[33]	$4x$	Souza Chies 91	BRA, RS, Piratini
[33]	$4x$	St s/n	BRA, RS, Eldorado do Sul

[15]	4x	V 10137, BRA-10006	BRA, SC, Laguna
[15]	4x	V 10629, BRA-10944	BRA, RS, Iraí
[15]	4x	V 10648, BRA-11011	BRA, RS, São Luiz Gonzaga
[15]	4x	V 11149, BRA-13374	BRA, PR, Quatro Barras
[15]	4x	V 11376, BRA-14206	BRA, PR, Guarapuava
[15]	4x	V 11387, BRA-14231	BRA, SC, São Lourenço do Oeste
[15]	4x	V 11388, BRA-14249	BRA, SC, São Lourenço do Oeste
[15]	4x	V 11414, BRA-14265	BRA, PR, Pato Branco
[15]	4x	V 11452, BRA-14401	BRA, PR, Palmas
[15]	4x	V 11538, BRA-14583	BRA, SC, Campos Novos
[15]	4x	V 11553, BRA-14656	BRA, SC, Curitibanos
[15]	4x	V 11664, BRA-12254	BRA, AP, Macapá
[15]	4x	V 11741, BRA-12491	BRA, MS, Três Lagoas
[15]	4x	V 11742, BRA-12505	BRA, MS, Três Lagoas
[15]	4x	V 11795, BRA-12564	BRA, MS, Sidrolândia
[15]	4x	V 11808, BRA-12637	BRA, MS, Caarapó
[15]	4x	V 11853, BRA-12815	BRA, MS, Iguatemi
[15]	4x	V 11883, BRA-12891	BRA, MS, Ponta Porã
[15]	4x	V 11914, BRA-13005	BRA, MS, Amambaí

[15]	4*x*	V 12121, BRA-16381	BRA, SC, São Joaquim
[15]	4*x*	V 12122, BRA-16390	BRA, SC, São Joaquim
[15]	4*x*	V 12156, BRA-16454	BRA, SC, Lages
[15]	4*x*	V 12160, BRA-16489	BRA, RS, Vacaria
[15]	4*x*	V 12161, BRA-16497	BRA, RS, Vacaria
[15]	4*x*	V 12164, BRA-16501	BRA, RS, Vacaria
[15]	4*x*	V 12178, BRA-16551	BRA, RS, Soledade
[15]	4*x*	V 12179, BRA-16560	BRA, RS, Soledade
[15]	4*x*	V 12203, BRA-16586	BRA, RS, Santa Barbara do Sul
[15]	4*x*	V 12208, BRA-16608	BRA, RS, Ijuí
[15]	4*x*	V 12234, BRA-16705	BRA, RS, S. Antônio das Missões
[15]	4*x*	V 12248, BRA-16781	BRA, RS, São Borja
[15]	4*x*	V 12253, BRA-16811	BRA, RS, Itaqui
[15]	4*x*	V 12260, BRA-16845	BRA, RS, Itaqui
[15]	4*x*	V 12267, BRA-16900	BRA, RS, Santiago
[15]	4*x*	V 12273, BRA-16926	BRA, RS, Santa Maria
[15]	4*x*	V 12274, BRA-16934	BRA, RS, Santa Maria
[15]	4*x*	V 12275, BRA-16942	BRA, RS, Santa Maria
[15]	4*x*	V 12286, BRA-17019	BRA, RS, São Sepé

[15]	4x	V 12293, BRA-17051	BRA, RS, Caçapava do Sul
[15]	4x	V 12324, BRA-17132	BRA, RS, Dom Pedrito
[15]	4x	V 12353, BRA-17248	BRA, RS, Santana do Livramento
[15]	4x	V 12359, BRA-17281	BRA, RS, Santana do Livramento
[15]	4x	V 12360, BRA-17299	BRA, RS, Santana do Livramento
[15]	4x	V 12361, BRA-17302	BRA, RS, Santana do Livramento
[15]	4x	V 12368, BRA-17345	BRA, RS, Santana do Livramento
[15]	4x	V 12369, BRA-17353	BRA, RS, Santana do Livramento
[15]	4x	V 12374, BRA-17396	BRA, RS, Quaraí
[15]	4x	V 12375, BRA-17400	BRA, RS, Quaraí
[15]	4x	V 12376, BRA-17418	BRA, RS, Quaraí
[15]	4x	V 12377, BRA-17436	BRA, RS, Quaraí
[15]	4x	V 12378, BRA-17434	BRA, RS, Quaraí
[15]	4x	V 12379, BRA-17442	BRA, RS, Quaraí
[15]	4x	V 12392, BRA-17512	BRA, RS, Uruguaiana
[15]	4x	V 12393, BRA-17523	BRA, RS, Uruguaiana
[15]	4x	V 12394, BRA-17531	BRA, RS, Uruguaiana
[15]	4x	V 12401, BRA-17582	BRA, RS, Uruguaiana
[15]	4x	V 12402, BRA-17591	BRA, RS, Uruguaiana

[15]	4*x*	V 12403, BRA-17604	BRA, RS, Uruguaiana
[15]	4*x*	V 12405, BRA-17612	BRA, RS, Uruguaiana
[34]	4*x*	V 12407	BRA, RS, Uruguaiana
[15]	4*x*	V 12408, BRA-17639	BRA, RS, Uruguaiana
[15]	4*x*	V 12410, BRA-17655	BRA, RS, Uruguaiana
[15]	4*x*	V 12411, BRA-17663	BRA, RS, Uruguaiana
[15]	4*x*	V 12412, BRA-17671	BRA, RS, Uruguaiana
[15]	4*x*	V 12415, BRA-17698	BRA, RS, Uruguaiana
[15]	4*x*	V 12426, BRA-17744	BRA, RS, São Luíz Gonzaga
[15]	4*x*	V 12448, BRA-17817	BRA, SC, Água Doce
[15]	4*x*	V 12472, BRA-18210	BRA, RS, Bagé
[15]	4*x*	V 12750, BRA-18350	BRA, RS, São Gabriel
[15]	4*x*	V 12751, BRA-18368	BRA, RS, São Gabriel
[15]	4*x*	V 12752, BRA-18376	BRA, RS, São Gabriel
[15]	4*x*	V 12753, BRA-18384	BRA, RS, São Gabriel
[18]	4*x*	V 14244A	BRA, RS, Uruguaiana
[25]	4*x*	V 14244B	BRA, RS, Uruguaiana
[18,25]	4*x*	V 14244E	BRA, RS, Uruguaiana
[18]	4*x*	V 14244H	BRA, RS, Uruguaiana

[25]	4*x*	V 14282	BRA, PR, Candói
[25]	4*x*	V 14287	BRA, PR, Candói
[18,25,29]	4*x*	V 14310	BRA, RS, Barra do Quarai
[18]	4*x*	V 14314, M8	BRA, MS, Itaquiraí
[18,25,29]	4*x*	V 14326	BRA, RS, Capivari do Sul
[18,25,30]	4*x*	V 14327, BRA - 021547	BRA, RS, Capivari do Sul
[18,25]	4*x*	V 14329, BRA - 021563	BRA, RS, Capivari do Sul
[18,25]	4*x*	V 14614, BRA - 023566	BRA, MS Japorã
[29]	4*x*	V 14783	BRA, RS, Vale do Sol, BR 286
[18]	4*x*	V 14827, M10	BRA, PR, Candói
[18]	4*x*	V 14828, M11	BRA, PR, Candói
[18]	4*x*	V 14865, M13	BRA, RS, Capivari do Sul
[18]	4*x*	V 14866	BRA, RS, Capivari do Sul
[18]	4*x*	V 14866, M14	BRA, RS, Capivari do Sul
[18]	4*x*	V 14869	BRA, RS, Capivari do Sul
[25]	4*x*	V 14870	BRA, RS, Capivari do Sul
[18]	4*x*	V 14870, M17	BRA, RS, Capivari do Sul
[25]	4*x*	V 14871	BRA, RS, Capivari do Sul
[18, 25,33]	4*x*	V 14921	BRA, RS, Quaraí

[18,25,33]	4*x*	V 14931	BRA, RS, Alegrete
[35]	4*x*	V 16774	BRA, Distrito Federal, Brasilia, Parque Ecologico Berbardo Sayã
[15]	4*x*	V 4077, BRA-7668	BRA, RS, Bom Jesus
[15]	4*x*	V 4093, BRA-7676	BRA, RS, Vacaria
[15]	4*x*	V 4121, BRA-7684	BRA, RS, Vacaria (cult.)
[15]	4*x*	V 4186, BRA-7731	BRA, RS, Tupanciretá
[15]	4*x*	V 4385, BRA-7773	BRA, RS, Ivoti
[15]	4*x*	V 4700, BRA-7820	BRA, RS, Uruguaiana
[15]	4*x*	V 4701, BRA-7838	BRA, RS, Uruguaiana
[15]	4*x*	V 4736, BRA-7854	BRA, RS, Santana do Livramento
[15]	4*x*	V 4752, BRA-7889	BRA, RS, Osório
[15]	4*x*	V 9667 BRA-6271,	BRA, RS, Uruguaiana
[15]	4*x*	V 9684, BRA-6301	BRA, RS, Uruguaiana
[15]	4*x*	V 9747, BRA-6467	BRA, RS, Alegrete
[15,32]	4*x*	V 9782, BRA - 006513	BRA, RS, Uruguaiana
[15]	4*x*	V 9822, BRA-6599	BRA, RS, Santo Ângelo
[15]	4*x*	V 9828, BRA-6629	BRA, RS, Santo Ângelo
[15]	4*x*	V 9829, BRA-6637	BRA, RS, Santo Ângelo
[15]	4*x*	V 9830, BRA-6645	BRA, RS, Santo Ângelo

[41]	4*x*	Pohl & Selva 12960	CR, Guanacaste, Cafias, Finca La Pacifica
[5,31]	4*x*	Q4181	CU, Indio Hatuey Experimental Station
[5,28,30-31]	4*x*	Q3775	MX, Tamaulipas
[5,29,30-31]	4*x*	PER-01, SV2893	PE, Departament of Cajamarca, Cajabamba, El Huayo, 2100 msm.
[36]	4*x*	H2528	PY. Dpto. Central, Ruta 2, 18 km antes de Caacupé
[5,31]	4*x*	PAR-01 N160	PY, Amambay, 25 km N of Pedro Juan Caballero.
[2]	4*x*	PI 121415	PY, From Jardfn Botanico, Santissima Trinidad.
[13]	4*x*	PI 404665	PY, Paraguarí, Chololó, 15 Km N Paraguari.
[13]	4*x*	PI 404667	PY, Central, Desarrollo Ganadero-Estancia, 10 Km E Caacupé.
[13]	4*x*	PI 404670	PY, Itapua, 12 Km S Bella Vista
[13]	4*x*	PI 404672	PY, Central, 50 Km W San Juan Bautista, 150 km south of Asuncion
[37]	4*x*	RIVAROLA 28	PY, Concepción, San Lázaro, Tres Cerros
[37]	4*x*	RIVAROLA 39	PY, Concepción, San Lázaro, Tres Cerros
[38]	4*x*	P.I. 158822	Paraguay, Central, Barrerito Ranch
[10,29]	4*x*	16N PI 276251	URU, Montevideo.
[10,29]	4*x*	37N PI 404861	URU, Santa Lúcia, Barra Rio Santa Lucia.
[10,29]	4*x*	69N PI 404866	URU, Rio Negro. East side of Rio Negro, 137 km east of Tacuarembó, on Route 26.
[10,29]	4*x*	89N PI 404865	URU, Tacuarembó. On Route 26, 102 km east-southeast of Tacuarembó.

[18]	4*x*	M66 MD S/N	URU, Colonia
[18,33]	4*x*	M67	URU, Colonia
[38]	4*x*	PI 155105	URU, Montevideo Campo Experimental de Pastos, Estancia Rincón de Santa Elena, Estación Alejandro Oallinal.
[13]	4*x*	PI 404861	URU, Montevideo, Rio Santa Lucia, 12 Km W Montevideo.
[13]	4*x*	PI 404864	URU, Paysandu, Route 26, 129 Km NE Paysandu.
[13]	4*x*	PI 404867	URU, Treinta Y Tres, Route 8, 8 Km SW
[39]	4*x*	URPN 24-2.	URU, Cerro Largo, Ruta 44, Km 10
[40]	4*x*	URPN003-2	URU, Salto, Route 31 km. 155.5, Carumbé
[40]	4*x*	URPN008-2	URU, Salto, Route 31 km. 16.1
[40]	4*x*	URPN010-4	URU, Salto, Route 3 km. 541.1
[40]	4*x*	URPN010-5	URU, Salto, Route 3 km. 541.1
[40]	4*x*	URPN014-1	URU, Rivera, Route 30 km. 228.5, Masoller
[40]	4*x*	URPN015-1	URU, Rivera, Route 5 km. 449
[40]	4*x*	URPN015-2	URU, Rivera, Route 5 km. 449
[39]	4*x*	URPN015-2.	URU, Rivera, Ruta 5, km 449
[40]	4*x*	URPN015-3	URU, Rivera, Route 5 km. 449
[40]	4*x*	URPN015-4	URU, Rivera, Route 5 km. 449
[40]	4*x*	URPN016-1	URU, Tacuarembó, Route 26 km. 271.8

[40]	4*x*	URPN019-1	URU, Tacuarembó, Route 26 km. 362.9, Caraguatá
[39]	4*x*	URPN019-1.	URU, Tacuarembo, Ruta 23, Km 362,9, Caraguata
[40]	4*x*	URPN023-2	URU, Cerro Largo, Route 44 km. 31.9
[40]	4*x*	URPN024-1	URU, Tacuarembó, Route 44 km. 64.9
[40]	4*x*	URPN024-2	URU, Tacuarembó, Route 44 km. 64.9
[40]	4*x*	URPN026-3	URU, Rivera, Route 27 km. 123.5, Vichadero
[40]	4*x*	URPN027-1	URU, Rivera, Road to Abrojal, 1 km from R27
[40]	4*x*	URPN032-1	URU, Tacuarembó, Route 5 km. 373.5, Bonilla
[40]	4*x*	URPN032-3	URU, Tacuarembó, Route 5 km. 373.5, Bonilla
[40]	4*x*	URPN041-1	URU, Tacuarembó, Route 59, passing Clara
[40]	4*x*	URPN041-3	URU, Tacuarembó, Route 59, passing Clara
[40]	4*x*	URPN045-1	URU, Paysandú, Route 3 km. 391.5
[40]	4*x*	URPN045-5	URU, Paysandú, Route 3 km. 391.5
[40]	4*x*	URPN053-1	URU, Durazno, Route 14 km. 166
[40]	4*x*	URPN053-3	URU, Durazno, Route 14 km. 166
[40]	4*x*	URPN053-4	URU, Durazno, Route 14 km. 166
[40]	4*x*	URPN054-3	URU, Durazno, Route 5 km. 187.5, Air Force
[40]	4*x*	URPN060-1	URU, Treinta y Tres, Route 8 km. 315.5
[40]	4*x*	URPN062-1	URU, Lavalleja, Route 14 km. 293.3

[40]	4*x*	URPN062-5	URU, Lavalleja, Route 14 km. 293.3
[40]	4*x*	URPN067-2	URU, Rocha, Route 9 km. 203.5
[40]	4*x*	URPN071-2	URU, Maldonado, Route Interbalnearia km. 87
[40]	4*x*	URPN074-1	URU, San José, Route 1 km. 66.6
[40]	4*x*	URPN076-1	URU, Colonia, Route 1 km. 141.7
[40]	4*x*	URPN076-2	URU, Colonia, Route 1 km. 141.7
[40]	4*x*	URPN076-3	URU, Colonia, Route 1 km. 141.7
[40]	4*x*	URPN076-4	URU, Colonia, Route 1 km. 141.7
[40]	4*x*	URPN078-1	URU, Colonia, Route 21 km. 204.8
[40]	4*x*	URPN081-1	URU, Colonia, Route 21 Est. Bella Vista
[40]	4*x*	URPN081-2	URU, Colonia, Route 21 Est. Bella Vista
[40]	4*x*	URPN081-3	URU, Colonia, Route 21 Est. Bella Vista
[40]	4*x*	URPN081-4	URU, Colonia, Route 21 Est. Bella Vista
[40]	4*x*	URPN084-4	URU, Soriano, Route 14 km. 29.9
[40]	4*x*	URPN086-1	URU, Flores, Route 14 km. 92.5
[39]	4*x*	URPN23-2.	URU, Cerro Largo, Ruta 44, Km 39,9
[39]	4*x*	URPN24-1.	URU, Cerro Largo, Ruta 44, Km 64,9
[39]	4*x*	URPN27-1.	URU, Rivera, Camino al Abrojal, 1 km de R27
[39]	4*x*	URPN27-2.	URU, Rivera, Camino al Abrojal, 1 km de R27

[39]	4*x*	URPN32-3	URU, Tacuarembó, Ruta 5, km 373,5, Bonilla
[39]	4*x*	URPN53-1	URU, Flores, Ruta 14, Km 166
[39]	4*x*	URPN62-1.	URU, Lavalleja, Ruta 14, KM 293,3
[39]	4*x*	URPN62-5.	URU, Lavalleja, Ruta 14, KM 293,3
[39]	4*x*	URPN67-2.	URU, Rocha, Ruta 9, KM 203,5
[39]	4*x*	URPN86-1	URU, Flores Ruta 14 km 92,5
This work	4*x*	H2137	ARG, Corrientes. Paso de los libres. Plaza central Bartolomé Mitre.
This work	4*x*	H2543 #1, #2, #3	ARG, Misiones, Eldorado, ruta provincial 17 a 11 km de Pozo Azul.
This work	4*x*	H2554 #1, #2, #3, #4, #5	ARG, Misiones, Capital, Garupá, al lado de estación de Trenes.
This work	4*x*	H2665	ARG, Misiones, Concepción de la Sierra, Ruta provincial 2.
This work	4*x*	H2671	ARG, Misiones, Ruta provincial 2, El Palmar del Río.
This work	4*x*	H2674 #1 - #11	ARG, Misiones, Panambí, Ruta provincial 2. Mar 2022.
This work	4*x*	H1271	ARG, Misiones, Guaraní, Arroyo Yerbas del Paraíso
This work	4*x*	H1350	ARG, Misiones, Capital, Bañados del Zaimán
This work	4*x*	H2295	BRA. PR. Route BR280, A 57 km de Francisco Beltrão.
This work	4*x*	H2304	BRA. SC, BR 116. 1,5 km S da prefeitura de Correia Pinto. [ca. V14411].
This work	4*x*	H2309	BRA. SC, BR116 de Urupema a Lages, near Lages.
This work	4*x*	H2310	BRA. SC, Correia Pinto, BR116 y Rua Duque de Caixas y Rua Cándido [ca. V14411].

This work	4*x*	H2312	BRA. SC BR116 de Urupema a Lages, near Lages.
This work	4*x*	H2319	BRA. SC, BR-116 a 50 km al N de Vacaría [ca. V12159].
This work	4*x*	H2691	BRA. RJ, Barra de Tijuca, na rua
This work	4*x*	H2693	BRA. RJ, Jardim Botânico Rio de Janeiro.
This work	4*x*	H3003	BRA. SC, BR116 KM210.
This work	4*x*	H3007 B	BRA. SC, BR116, 5 km N Rio Canoas. *Honfi et al. 3007* (MNES). [ca. V8211]
This work	4*x*	H3008 C	BRA. SC, BR116, 5 km N Rio Canoas.
This work	4*x*	H3016	BRA. SC, BR116 Correia Pinto.
This work	4*x*	H2528	PY. Central. Sobre Ruta nacional 2, a 18 km de Caacupé.
This work	4*x*	Ch – 1	PY. Compañía Capilla cué, pastizal. Abr 2022. *Chaparro 1* (FACEN).
This work	4*x*	Ch – 2	PY. Compañía Capilla cué, pastizal. Abr 2022. *Chaparro 2* (FACEN).
This work	4*x*	Ch – 3	PY. Compañía Capilla cué, pastizal. Abr 2022. *Chaparro 3* (FACEN). S -25.559465, W -57.084862
This work	4*x*	Ch – 4	PY. Compañía Capilla cué, pastizal, costado de camino calle Faustino Rivas. Abr 2022. *Chaparro 4* (FACEN).
This work	4*x*	Ch – 5	PY. Compañía Capilla cué, pastizal costado de camino calle Faustino Rivas. Abr 2022. *Chaparro 5* (FACEN).
This work	4*x*	Ch – 6	PY. Compañía Capilla cué, pastizal, costado de camino calle Faustino Rivas. Abr 2022. *Chaparro 6* (FACEN).
This work	4*x*	Ch – 7	PY. Compañía Capilla cué, pastizal. Abr 2022. *Chaparro 7* (FACEN).
This work	4*x*	Ch – 8	PY. Compañía Capilla cué, pastizal. Abr 2022. *Chaparro 8* (FACEN).
This work	4*x*	Ch – 9	PY. Compañía Capilla cué, pastizal. Abr 2022. *Chaparro 9* (FACEN).

This work	4*x*	Ch – 10	PY. Compañía Capilla cué, pastizal. Abr 2022. *Chaparro 10* (FACEN).
This work	4*x*	Ch – 11	PY. Compañía Capilla cué, pastizal. Abr 2022. *Chaparro 11* (FACEN).
This work	4*x*	Ch – 12	PY. Compañía Capilla cué, pastizal. Abr 2022. *Chaparro 12* (FACEN). S -25.569693, W -57.063805
This work	4*x*	Ch – 13	PY. Compañía Capilla cué, pastizal. Abr 2022. *Chaparro 13* (FACEN).
This work	4*x*	Ch – 14	PY. Compañía Capilla cué, pastizal. Abr 2022. *Chaparro 14* (FACEN).
This work	4*x*	Ch – 25	PY. Central. Ciudad de Luque, costado de camino. Abr 2022. *Chaparro 25* (FACEN).
This work	4*x*	Ch – 26	PY. Central. Luque, paseo central, ruta. Abr 2022. *Chaparro 26* (FACEN).
This work	4*x*	Ch - 27	PY. Central. Luque, paseo central, ruta. Abr 2022. *Chaparro 27* (FACEN).
This work	4*x*	Ch - 29	PY. Central. Luque, costado de camino. Abr 2022. *Chaparro 29* (FACEN).
This work	4*x*	Ch - 31	PY. Paraguarí. Ruta PY 01, costado de camino. Abr 2022. *Chaparro 31* (FACEN).
This work	4*x*	Ch - 33	PY. Paraguarí. Ruta PY 01, costado de camino. Abr 2022. *Chaparro 33* (FACEN).
This work	4*x*	Ch – 33B	PY. Paraguarí. Ruta PY 01, costado de camino. Abr 2022. *Chaparro 33B* (FACEN).
This work	4*x*	Ch - 34	PY. Paraguarí. Ruta PY 01, costado de camino. Abr 2022. *Chaparro 34* (FACEN).
This work	4*x*	Ch - 35	PY. Paraguarí. Ruta PY 01, costado de camino. Abr 2022. *Chaparro 35* (FACEN).
This work	4*x*	Ch - 36	PY. Paraguarí. Ruta PY 01, costado de camino. Abr 2022. *Chaparro 36* (FACEN).
This work	4*x*	Ch - 37	PY. Paraguarí. Ruta PY 01, costado de camino. Abr 2022. *Chaparro 37* (FACEN).
This work	4*x*	Ch - 38	PY. Paraguarí. Ruta 01, costado de camino, frente al cerro Yaguarón. Abr 2022. *Chaparro 38* (FACEN).

This work	*4x*	Ch - 40	PY. Paraguarí. Ruta 01, costado de camino, frente al cerro Yaguarón. Abr 2022. *Chaparro 40* (FACEN).
This work	*4x*	Ch - 41	PY. Paraguarí. Ruta 01, ciudad de Paraguarí, costado de camino. Abr 2022. *Chaparro 41* (FACEN).
This work	*4x*	Ch - 42	PY. Paraguarí. Ruta 01, ciudad de Paraguarí, costado de camino. Abr 2022. *Chaparro 42* (FACEN).
This work	*4x*	Ch - 43	PY. Paraguarí. Ruta PY 01, Paraguarí, costado de camino. Abr 2022. *Chaparro 43* (FACEN).
This work	*4x*	Ch - 44	PY. Paraguari. Ruta PY 01, d de Paraguari, costado de camino. Abr 2022. *Chaparro 44* (FACEN).
This work	*4x*	Ch - 45	PY. Paraguarí. Ruta PY 01, ciudad de Paraguari, costado de camino. Abr 2022. *Chaparro 45* (FACEN).
This work	*4x*	Ch - 47	PY. Presidente Hayes. Ruta transchaco, costado de camino. Abr 2022. *Chaparro 47* (FACEN).
This work	*4x*	Ch - 48	PY. Presidente Hayes. Ruta transchaco, costado de camino. Abr 2022. *Chaparro 48* (FACEN). S -25.134778, W -57.555056
This work	*4x*	Ch - 49	PY. Presidente Hayes. Ruta transchaco, costado de camino Abr 2022. *Chaparro 49* (FACEN).
This work	*4x*	Ch - 50	PY. Presidente Hayes. Ruta transchaco, costado de camino03/04/2022. (FACEN).
This work	*4x*	Ch - 51	PY. Dto. Presidente Hayes. Ruta transchaco, costado de camino. Abr 2022. *Chaparro* (FACEN).
This work	*4x*	Ch - 52	PY. Dto. Presidente Hayes. Ruta transchaco, costado de camino. Abr 2022. *Chaparro 52* (FACEN).
This work	*4x*	Ch - 53	PY. Presidente Hayes. Ruta transchaco, costado de camino. Abr 2022. *Chaparro 53* (FACEN).
This work	*4x*	Ch - 54	PY. Presidente Hayes. Ruta transchaco, costado de camino. Abr 2022. *Chaparro 54* (FACEN).
This work	*4x*	Ch - 55	PY. Presidente Hayes. Ruta transchaco, costado de camino. Abr 2022. *Chaparro 55* (FACEN).

This work	*4x*	Ch - 56	P. Presidente Hayes. Ruta transchaco, costado de camino. Abr 2022. *Chaparro 56* (FACEN).
This work	*4x*	Ch - 57	PY. Presidente Hayes. Ruta transchaco, costado de camino. Abr 2022. *Chaparro 57* (FACEN).
This work	*4x*	Ch - 58	PY. Presidente Hayes. Ruta transchaco, costado de camino. Abr 2022. *Chaparro* (FACEN).
This work	*4x*	Ch- 59	PY. Presidente Hayes. Ruta transchaco, costado de camino. Abr 2022. *Chaparro 59* (FACEN).
This work	*4x*	Ch - 60	PY. Presidente Hayes. Ruta transchaco, costado de camino. Abr 2022. *Chaparro 60* (FACEN).
This work	*4x*	Ch - 61	PY. Presidente Hayes. Ruta transchaco, costado de camino. Abr 2022. *Chaparro 61* (FACEN).
This work	*4x*	H2690	BRA. Barra da Tijuca, Rio de Janeiro. Ene 2018. *Honfi 2690RB #8* (MNES).
[13]	5*x*	PI 508825	BRA, SC, Near Route BR 116, 46 Km N Lages On hill off Route BR 116, 46km N of Lages, Santa Catarina.
This work	5*x*	H3007 B	BRA, SC BR116, 5 km N Rio Canoas. [near V8211]
[5]	6*x*	Q4131	ARG, Santa Fe, Santa Fe, 15km W of La Criolla

References: ARG, Argentina; BRA, Brazil, BO, Bolivia; CR, Costa Rica; CU, Cuba; MX, Mexico; PE, Peru; PY, Paraguay; URU, Uruguay; USA, United State of America. RS, Rio Grande do Sul; SC, Santa Catarina; PR, Parana; SP, São Paulo.
x: ploidy level. 2*x*; diploids (2*n*= 20); 3*x*; triploids (2*n*= 30); 4*x*; tetraploids (2*n*= 40); 5*x*; pentaploids (2*n*= 50); 6*x*; hexaploids (2*n*= 60) chromosomes.

References

1. Saura, F. Cariología de gramíneas en Argentina. *Rev. Fac. Agron. Vet. B. Aires* **1948,** *12*, 51-67.
2. Burton, G.W. A cytological study of some species in the genus *Paspalum. J. Ag. Res*., **1940**, 3, 193-198.
3. Quarin, C.L. Recuentos cromosómicos en gramíneas de Argentina subtropical. *Hickenia* **1977,** *1,* 73-78. https://www.hickenia.darwin.edu.ar/index.php/hickenia/article/view/98

4. Martínez, E.J.; Espinoza, F.; Quarin, C.L. BIII progeny (2*n* + *n*) from apomictic *Paspalum notatum* obtained through early pollination. *J. Hered.* **1994**, *85*(4), 295-297. https://doi.org/10.1093/oxfordjournals.jhered.a111460
5. Espinoza, F.; Daurelio, L.D.; Pessino, S.C.; Valle, E.M.; Quarin, C.L. Genetic characterization of *Paspalum notatum* accessions by AFLP markers. *Plant Syst. Evol.* **2006,** 258, 147–159. https://doi.org/10.1007/s00606-005-0401-x
6. Espinoza F.; Quarin C.L. 2*n*+*n* hibridization of apomictic *Paspalum dilatatum* with diploid *Paspalum* species. *Int. J. Plant Sci.*, **2000,** 161, (2), 221-225.
7. Galdeano, F.; Urbani, M.H.; Sartor, M.E.; Honfi, A.I.; Espinoza, F.; Quarin, C.L. Relative DNA content in diploid, polyploid, and multiploid species of Paspalum (Poaceae) with relation to reproductive mode and taxonomy. *J. Pl. Res.* **2016**, *129*, 697-710. https://doi.org/10.1007/s10265-016-0813-4
8. Martínez, E.J.; Acuña, C.A.; Hojsgaard, D.H.; Quarin, C.L. Segregation for asexual seed production in *Paspalum* achieved by male gametes of apomictic triploid plants. *Ann. Bot.* **2007**, 100(6), 1239-1247. https://doi.org/10.1093/aob/mcm201
9. D'Aurelio, L.D.; Espinoza, F.; Quarin, C.L.; Pessino, S.C. Genetic diversity in sexual diploid and apomictic tetraploid populations of *Paspalum notatum* situated in sympatry or allopatry. *Plant Syst. Evol.* **2004**, *244*, 189-199. https://doi.org/10.1007/s00606-003-0070-6
10. Fachinetto J.M; Schneider R.; Hubber K.G.C.; Dall'Agnol M. Avaliação agronômica e análise da persistência em uma coleção de acessos de *Paspalum notatum* Flüggé (Poaceae). *Agrária* **2012,** 7, 189-195.
11. eutemann, A.V.; Rua, G.H.; Daviña, J.R.; Honfi, A.I. Poaceae. IAPT chromosome data 31/11. *In* Marhold, K. & Kucera, J. (eds.) & al., IAPT chromosome data 31. *Taxon* **2019**, 68, 1379–1380, E39–E42. [CrossRef]
12. Honfi, A.I.; Reutemann A.V.; Schneider, J.S.; Escobar, L.M.; Martínez, E.J.; Daviña, J.R. Chromosome Morphology and Heterochromatin Patterns in Paspalum notatum: Insights into Polyploid Genome Structure. *Genes* **2025**, 16, 242. https://doi.org/10.3390/genes16030242
13. Tischler, C.R.; Burson, B.L. Evaluating different bahiagrass cytotypes for heat tolerance and leaf epicular wax content. *Euphytica* **1995**, *84*, 229–235. https://doi.org/10.1007/BF01681815
14. Vega, J.M.; Podio, M.; Orjuela, J.; Siena, L.A.; Pessino, S.C.; Combes, M.C.; Mariac, C.; Albertini, E.; Pupilli, F.; Ortiz, J.P.A.; Leblanc, O. Chromosome-scale genome assembly and annotation of *Paspalum notatum* Flüggé var. *saurae*. *Sci. Data* **2024,** *11*, 891. https://doi.org/10.1038/s41597-024-03731-0
15. Pozzobon, M.T.; Valls, J.F.M. Chromosome number in germplasm accessions of *Paspalum notatum* (Gramineae). *Braz. J. Genet.* **1997**, *20*(1), 29-34. https://doi.org/10.1590/S0100-84551997000100006
16. Pagliarini M.S.; Carraro L.R.; de Freitas P.M.; Adamowski E.D.V.; Rocha Batista L.A.; Valls J.F.M. Cytogenetic characterization of Brazilian *Paspalum* accessions. *Hereditas* **2001** 135:27–34. https://doi:10.1111/j.1601-5223.2001.00027.x
17. Pagliarini M.S.; Takayama S.Y.; Freitas P.M.; Carraro L.R.; Adamowski E.V.; Batista L.A.R. Failure of cytokinesis and 2*n* gamete formation in Brazilian accessions of *Paspalum*. *Euphytica* **1999**, 108, 129-135.
18. Dahmer, N.; Schifino-Wittmann, M.T.; Dall'Agnol, M.; Castro, B. Cytogenetic data for *Paspalum notatum* Flugge accessions. *Sci. Agric.* **2008**, *65*, 381-388. https://doi.org/10.1590/S0103-90162008000400009
19. Burson, B.L.; Bennett H.W. Cytogenetics of *Paspalum urvillei* x *P. jurgensii* and *P. urvillei* x *P. vaginatum* hybrids. *Crop Sci.* **1972,** 12:105-108.

20. Burson, B.L.; Lee H.; Bennett H.W. Genome relations between tetraploid *Paspalum dilatatum* and four diploid *Paspalum* species. *Crop Sci.*, **1973**, 13, 739-743.
21. Burson, B.L. Genome Relations Among Four Diploid *Paspalum* Species. Bot. Gaz., **1981**, 142(4), 592-596.
22. Hojsgaard, D.H.; Honfi, A.I.; Rua, G.; Daviña, J.R. Chromosome numbers and ploidy levels of *Paspalum* species from subtropical South America (Poaceae) *Genet. Resour. Crop Evol.* **2009**, *56*, 533-545. https://doi.org/10.1007/s10722-008-9384-0
23. Rua, G.H.; Speranza, P.R.; Vaio, M.; Arakaki, M. A phylogenetic analysis of the genus *Paspalum* Poaceae based on cpDNA and morphology. *Pl Syst &Evol* **2010**, 288, 3-4, 227–243.
24. Catanzaro, M.P.; Bonasora, M.G.; Speranza, P.R.; Medina N.M.; Valls, J.F.M.; Rua, G.H. *Paspalum chilense* Poaceae, Paspaleae: A new species from southern South America. *Phytotaxa*, **2015**, 197 (4), 245–256.
25. Cidade, F.W.; Vigna, B.B.Z.; de Souza, F.H.D.; Valls, J.F.M.; Dall'Agnol, M.; Zucchi, M.I.; Souza-Chies T.T.; Souza A.P. Genetic variation in polyploid forage grass: assessing the molecular genetic variability in the *Paspalum* genus. *BMC Genet.*, **2013**, 14, 50. https://doi: 10.1186/1471-2156-14-50
26. Quarin C.L.; Norrmann G.A.; Urbani M.H. Polyploidization in aposporous *Paspalum* species. *Apomixis Newsl.* **1989**, 2, 44-46.
27. Gould, F.W. Chromosome numbers of Mexican grasses. *Can. J. Bot.* **1966**, *44*, 1683-1696. https://doi.org/10.1139/b66-181
28. Martínez, E.J.; Urbani, M.H.; Quarin, C.L.; Ortiz, J.P.A. Inheritance of apospory in bahiagrass, *Paspalum notatum*. *Hereditas*, **2001**, *135*, 19-25. https://doi.org/10.1111/j.1601-5223.2001.00019.x
29. Fachinetto, J.M.; Dall'agnol, M.; Schifino-Wittmann, M.T., Simioni, C.; Ávila, M.R. New wild diploids in *Paspalum notatum* Flügge (Poaceae): Potential accessions for use in breeding. *Crop Breed. Appl. Biotechnol.* **2018**, *18*(4), 432–436. https://doi.org/10.1590/1984-70332018v18n4n63
30. Zilli, A.L.; Acuña, C.A.; Schulz, R.R.; Brugnoli, E.A.; Guidalevich, V.; Quarin, C.L.; Martínez, E.J. Widening the gene pool of sexual tetraploid Bahiagrass: Generation and reproductive characterization of a sexual synthetic tetraploid population. *Crop Sci.* **2018**, *58*, 762-772. http://doi.org/10.2135/cropsci2017.07.0457
31. Rebozzio, R.N.; Sartor, M.E.; Quarin, C.L.; Espinoza, F. Residual sexuality and its seasonal variation in natural apomictic *Paspalum* notatum accessions. *Biologia Plantarum* **2011**, 55, 391–395.
32. Adamowski, E.V.; Pagliarini, M.S.; Bonato, A.B.M.; Batista, L.A.R.; Valls, J.F.M. Chromosome numbers and meiotic behavior of some *Paspalum* accessions. *Genet Mol Biol* **2005**, 28, 773–780.
33. Cidade, F.W.; Dall'Agnol, M.; Bered, F.; de Souza-Chies, T.T. Genetic diversity of the complex *Paspalum notatum* Flüggé (Paniceae: Panicoideae). *Genet. Resour. Crop Evol.* **2008**, *55*, 235–246. https://doi.org/10.1007/s10722-007-9231-8
34. Honfi A.I.; Quarin C.L.; Valls J.F.M. Estudio Cariológicos en Gramíneas Sudamericanas. Darwiniana **1990**, 30, 87-94.
35. Pozzobon, M.T.; dos Santos Sousa M.W.; Valls, J.F.M. Chromosome numbers of *Paspalum* species. *In*: Marhold & Kucera (eds.) IAPT chromosome data 36/4, *Taxon* **2022**, *71*, *(5)*, E13 – E18. https://doi.org/10.1002/tax.12809
36. Chaparro, C.; Escobar, L.M.; Schneider, J.S.; Eckers, F.; Perichon, M.C.; Daviña, J.R.; Honfi, A.I. Niveles de ploidía de algunas especies de *Paspalum* L. de Paraguay. *Steviana*, **2023**, 15 (1), 25-36. https://doi.org/10.56152/StevianaFacenV15N1A3_2023

37. Rivarola A.; Daviña, J.R.; Honfi A.I. Poaceae. *In*: Marhold, K. (ed.), IAPT/IOPB chromosome data 23. *Taxon*, **2016**, 55, 1457. https://doi.org/10.12705/656.34
38. Forbes, I.J.R.; Burton, G.W. Cytology of diploids, natural and induced tetraploids, and intra-species hybrids of Bahiagrass, *Paspalum notatum* Flügge. *Crop Sci*. **1961**, *1*(6), 402-406. https://doi.org/10.2135/cropsci1961.0011183X000100060006x.
39. Suarez V.C. *Paspalum notatum* Flüggé (Poaceae): estudio comparativo del comportamiento cromosómico em meiosis y tamaño y viabilidad de los granos de polen em dos grupos de diferente contenido de ADN. Tesis de grado **2014**, FCs-UDELAR, Montevideo, Uruguay, 79pp.
40. Reyno, R.; Narancio, R.; Speranza, P.; Do Canto, J.; López-Carro, B.; Hernández, P.; Burgueño, J.; Real, D.; Dalla Rizza, M. Molecular and cytogenetic characterization of a collection of bahiagrass (*Paspalum notatum* Flüggé) native to Uruguay. *Genet. Resour. Crop Evol*. **2012**, *59*,1823–1832. http://doi.org/10.1007/s10722-012-9806-x
41. Davidse G.; Pohl R.W. Chromosome numbers of Tropical American grasses (Gramineae). Ann. Missouri Bot. Gard., **1978,** 65, 637-649.
