# Supplementary figures and images for: Neonative Diploid-Polyploid Hotspots of Paspalum notatum: Identifying Novel Genetic Diversity for Conservation in South America
Source: Genes (Basel). 2025 Sep 16;16(9):1098. doi: 10.3390/genes16091098 (PMC12470053; doi:10.3390/genes16091098)

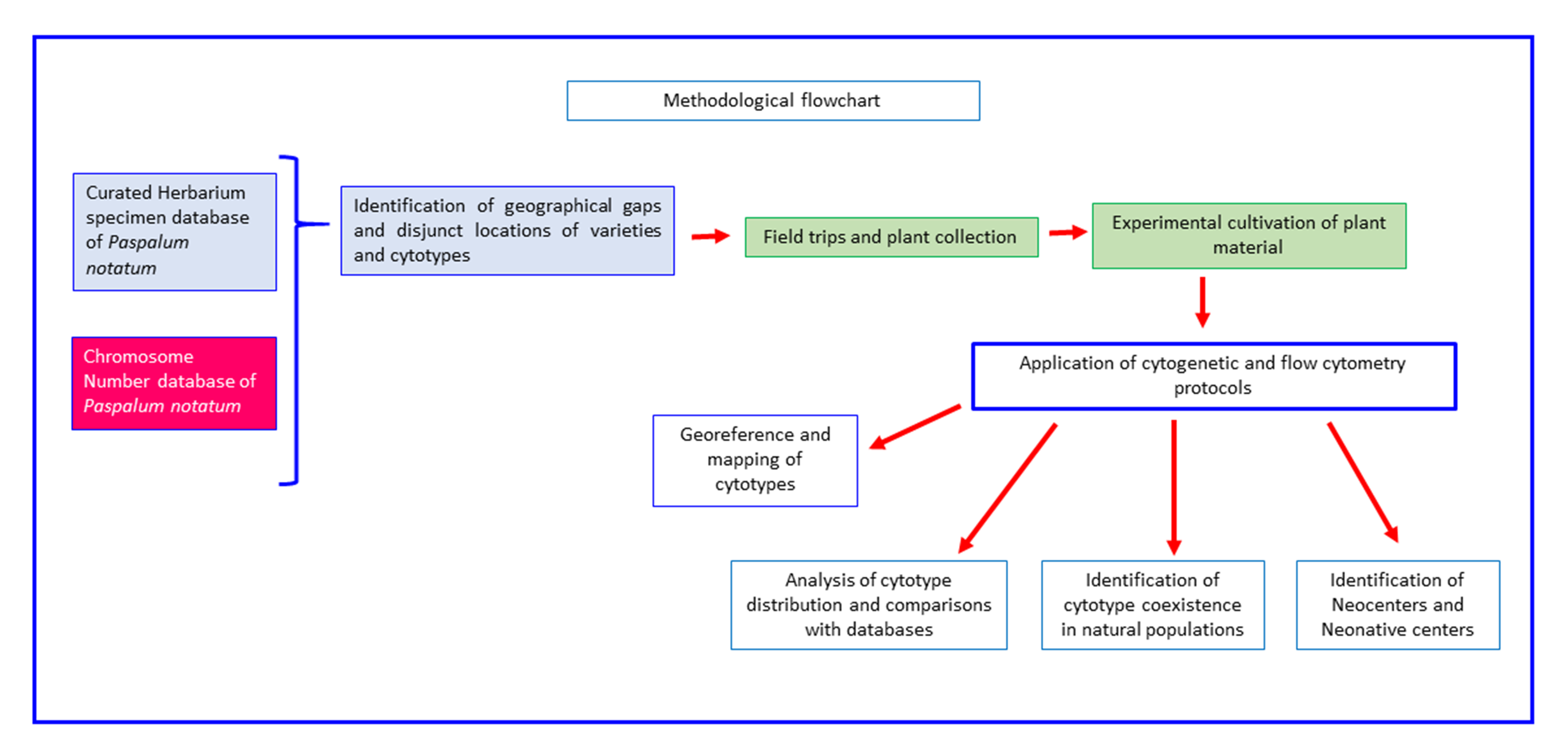

Supplement: Supplementary file 1 [file genes-16-01098-s001.zip › genes-3780646 manuscript revised FIGURE S1 Methodological flow chart VF.tif]
